# Supplementary material for: Transformable masks for colloidal nanosynthesis
Source: Nat Commun. 2018 Feb 8;9:563. doi: 10.1038/s41467-018-02958-x (PMC5805779; doi:10.1038/s41467-018-02958-x)
Supplement: Supplementary file 1 — Supplementary Information [file 41467_2018_2958_MOESM1_ESM.pdf]

Supplementary Information

Transformable Masks for Colloidal Nanosynthesis

Zhenxing Wang, Bowen He, Gefei Xu, Guojing Wang, Jiayi Wang, Yuhua Feng, Dongmeng Su, Bo Chen, Hai Li, Zhonghua Wu, Hua Zhang, Lu Shao, and Hongyu Chen

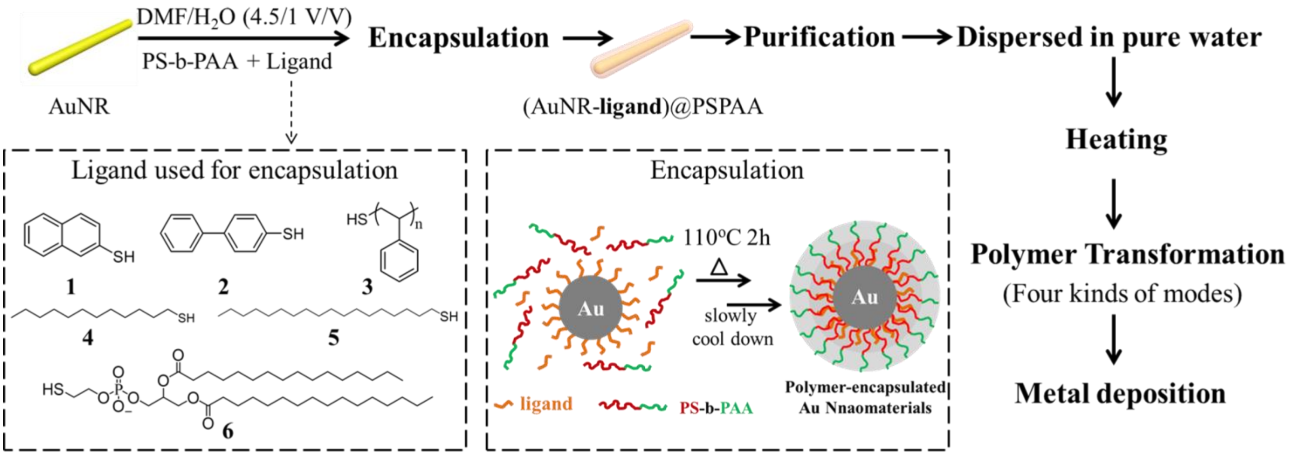

Supplementary Figure 1. Scheme showing the processes for the preparation of (AuNR-**ligand**)@PSPAA, transformation of PSPAA and metal deposition.

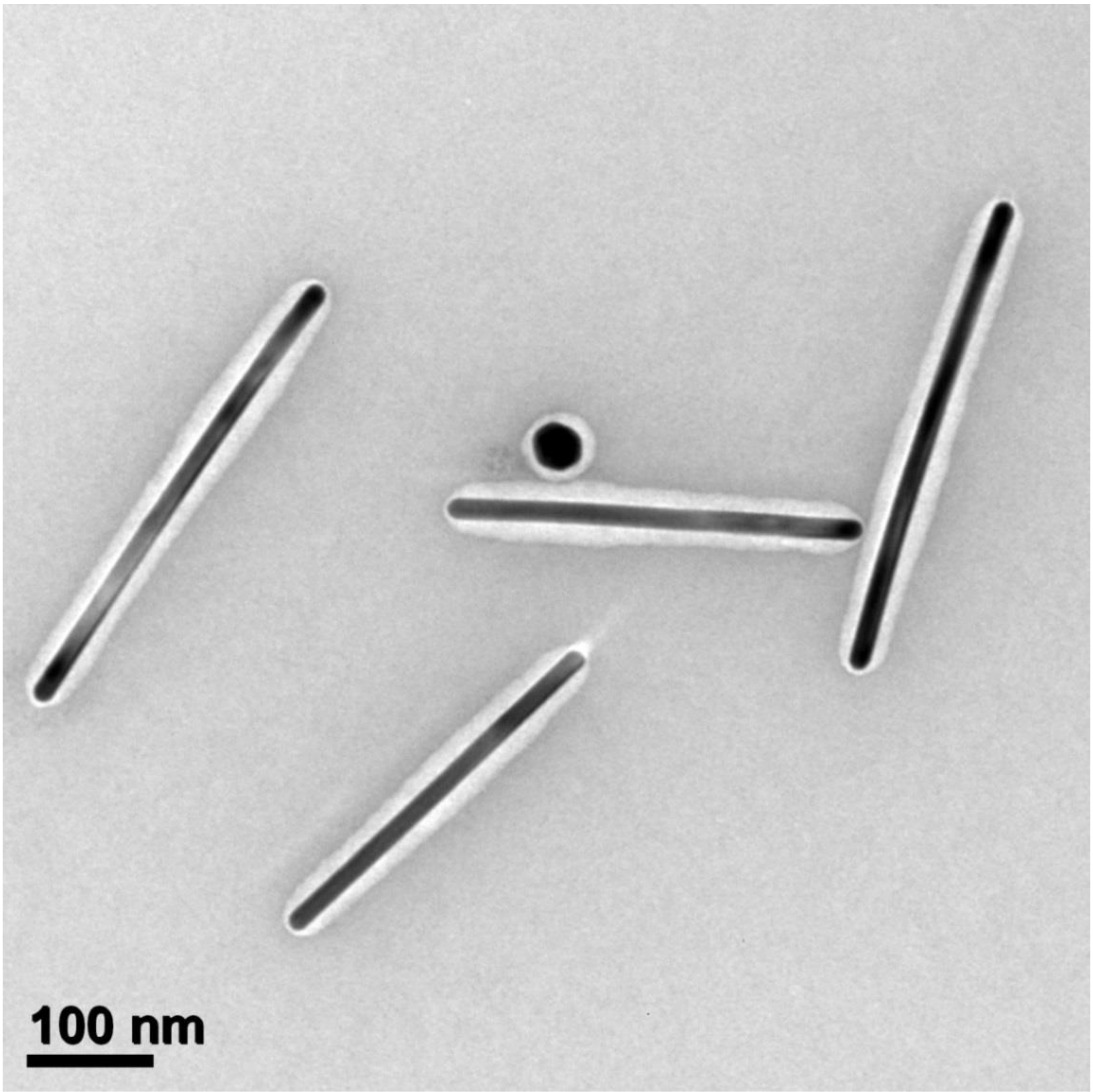

Supplementary Figure 2. TEM image of the pristine core-shell structure of (AuNR-**1**)@PSPAA.

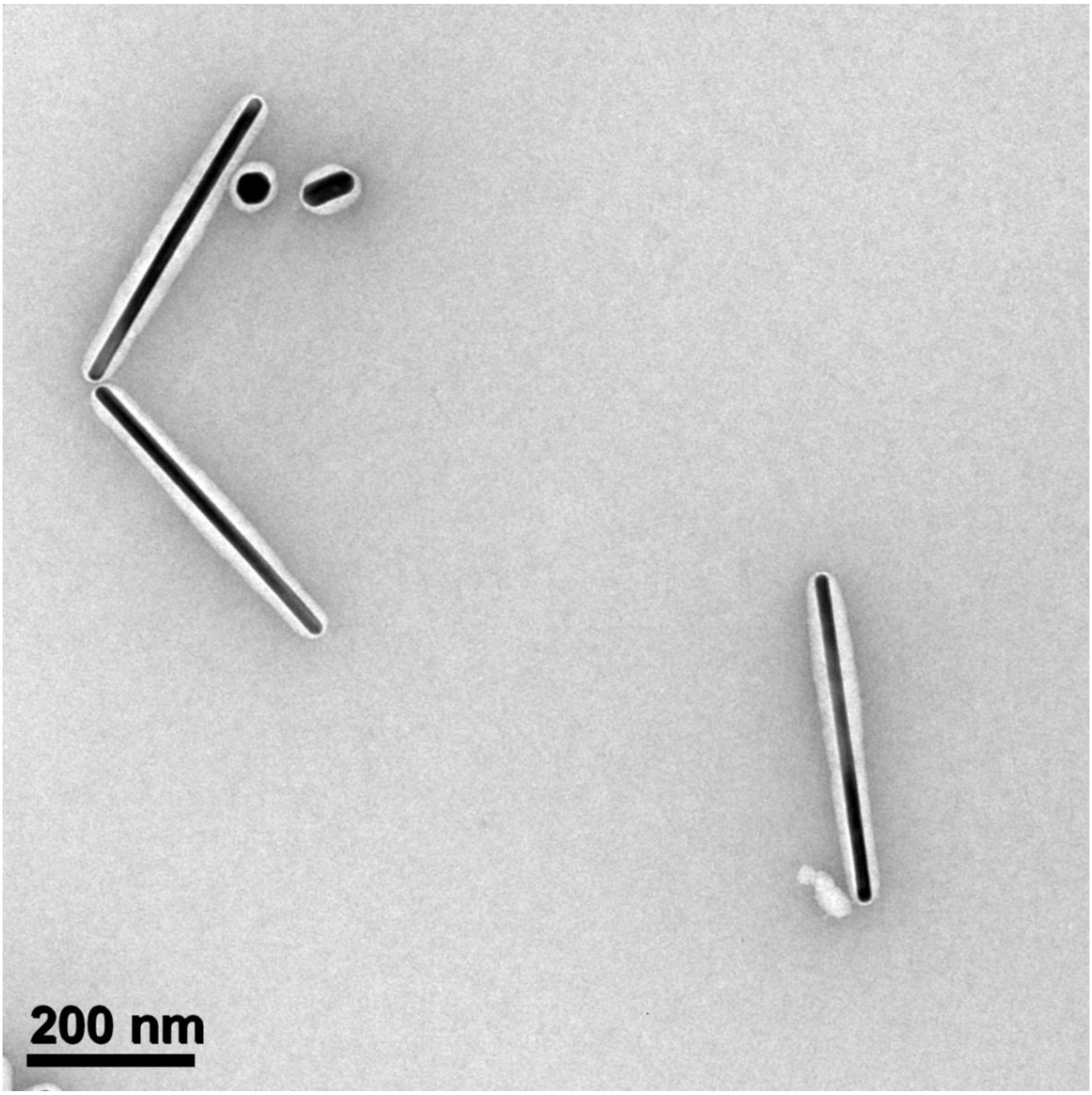

Supplementary Figure 3. TEM image of the pristine core-shell structure of (AuNR-**2**)@PSPAA.

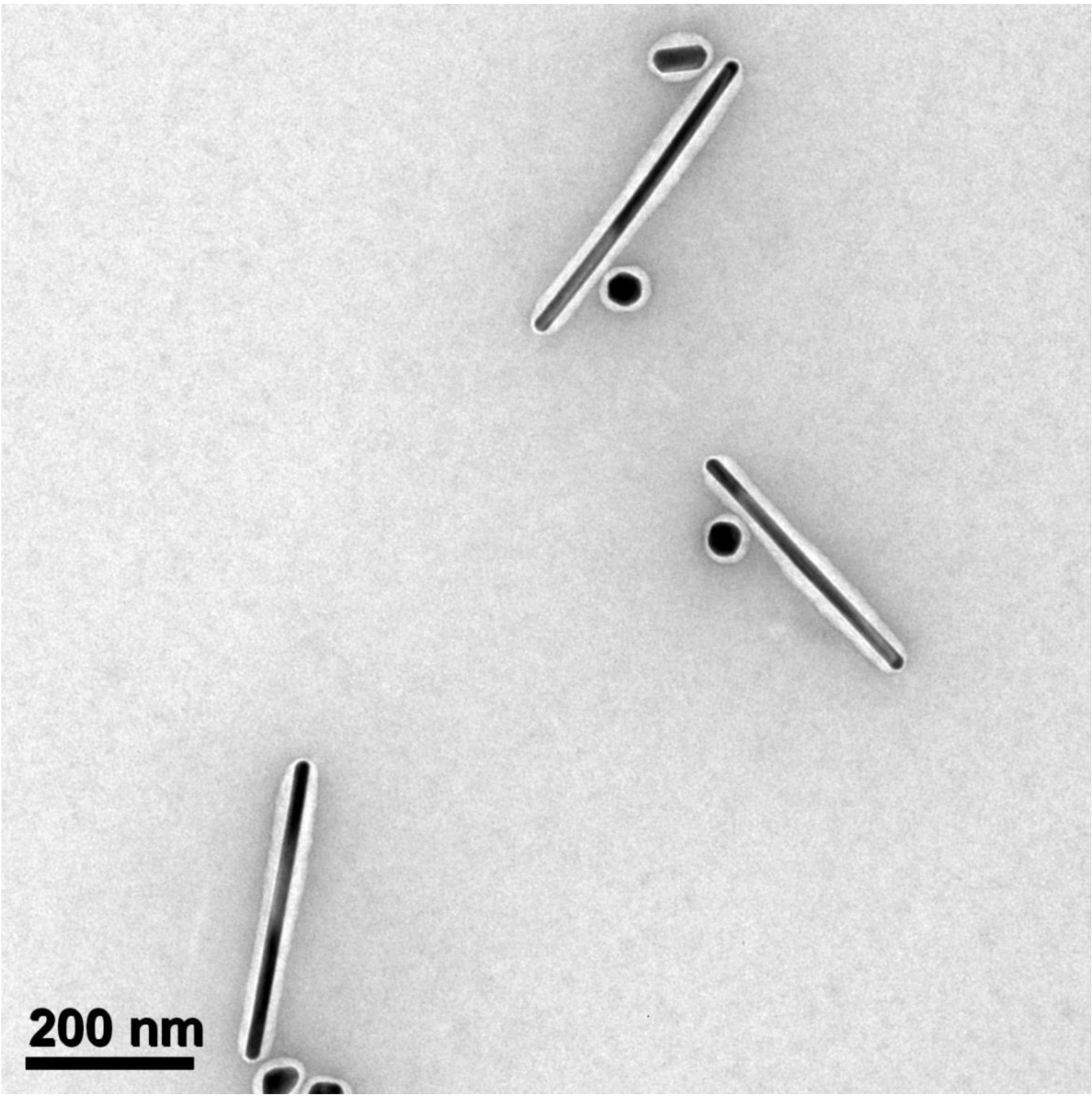

Supplementary Figure 4. TEM image of the pristine core-shell structure of (AuNR-**3**)@PSPAA.

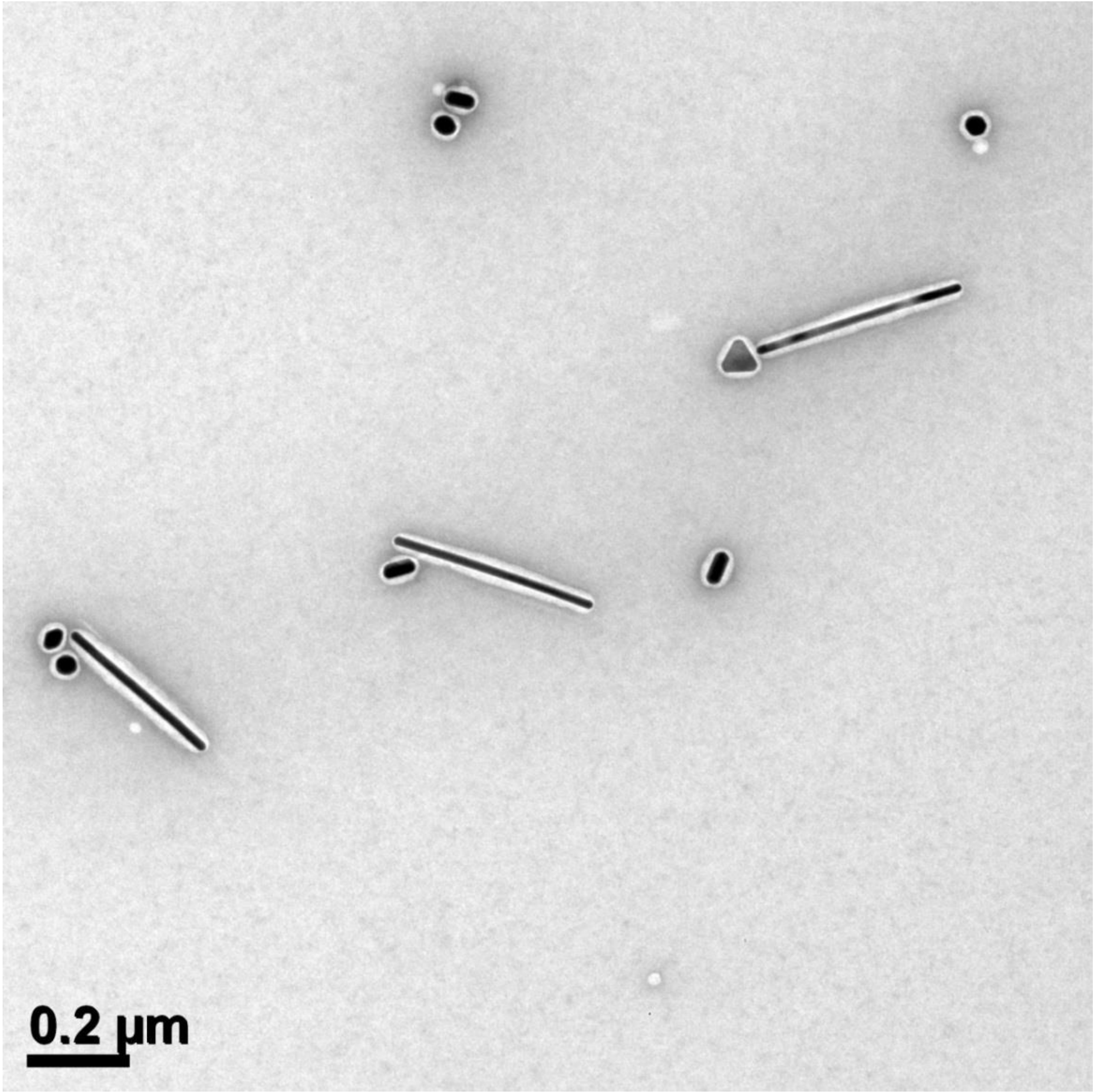

Supplementary Figure 5. TEM image of the pristine core-shell structure of (AuNR-**6**)@PSPAA.

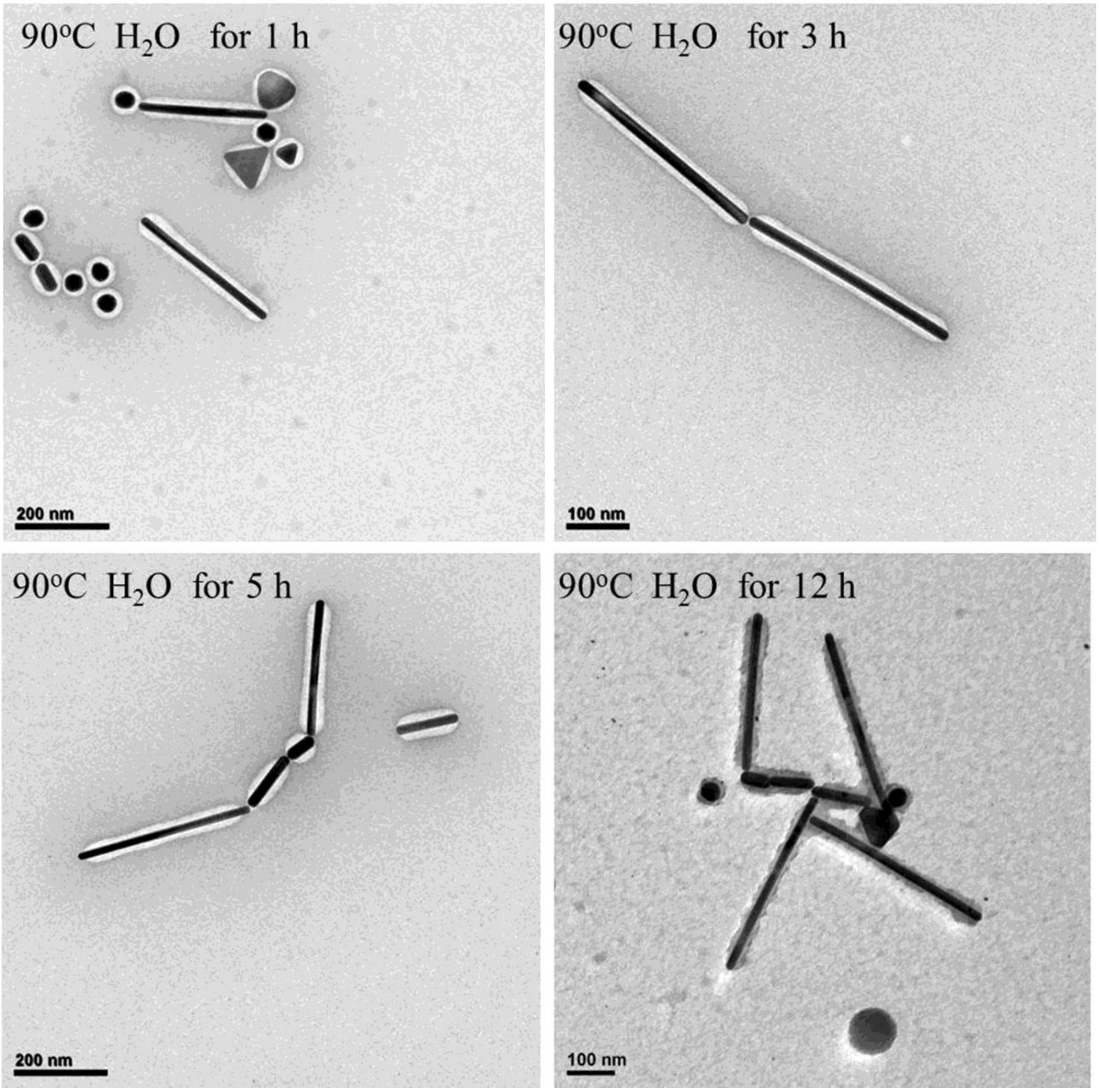

Supplementary Figure 6. TEM images of (AuNR-**1**)@PSPAA after being heated in water at 90 °C for 1 h, 3 h, 5 h and 12 h, respectively. (The ligand-**1** concentration in the encapsulation step is about 0.15 mM).

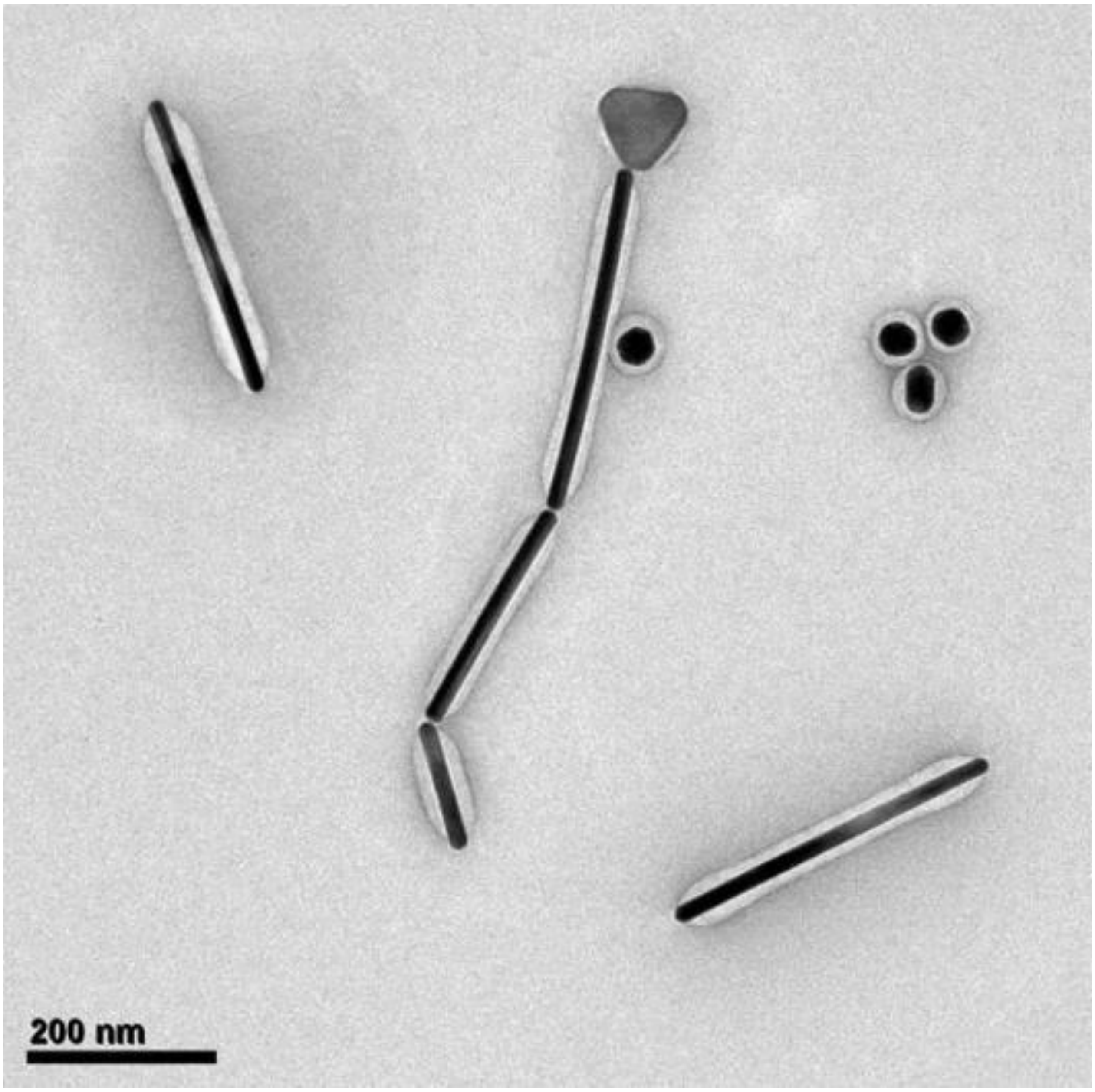

Supplementary Figure 7. TEM image of (AuNR-**1**)@PSPAA after being heated in water at 105 °C for 1.5 h. (The ligand-**1** concentration in the encapsulation step is about 0.15 mM).

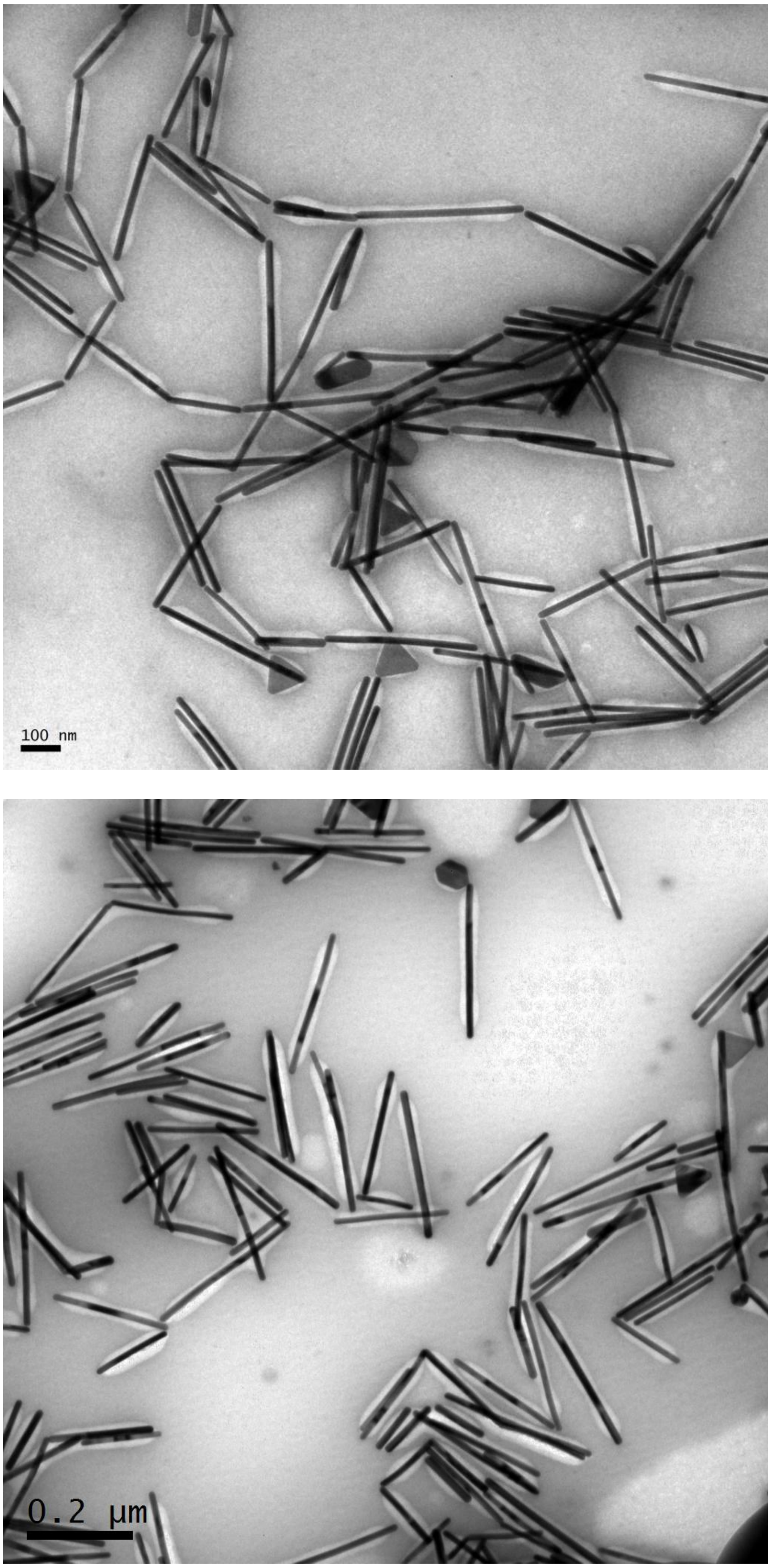

Supplementary Figure 8. TEM images of (AuNR-**1**)@PSPAA after being heated in water at 105 °C for 2.5 h. (The ligand-**1** concentration in the encapsulation step is about 0.15 mM).

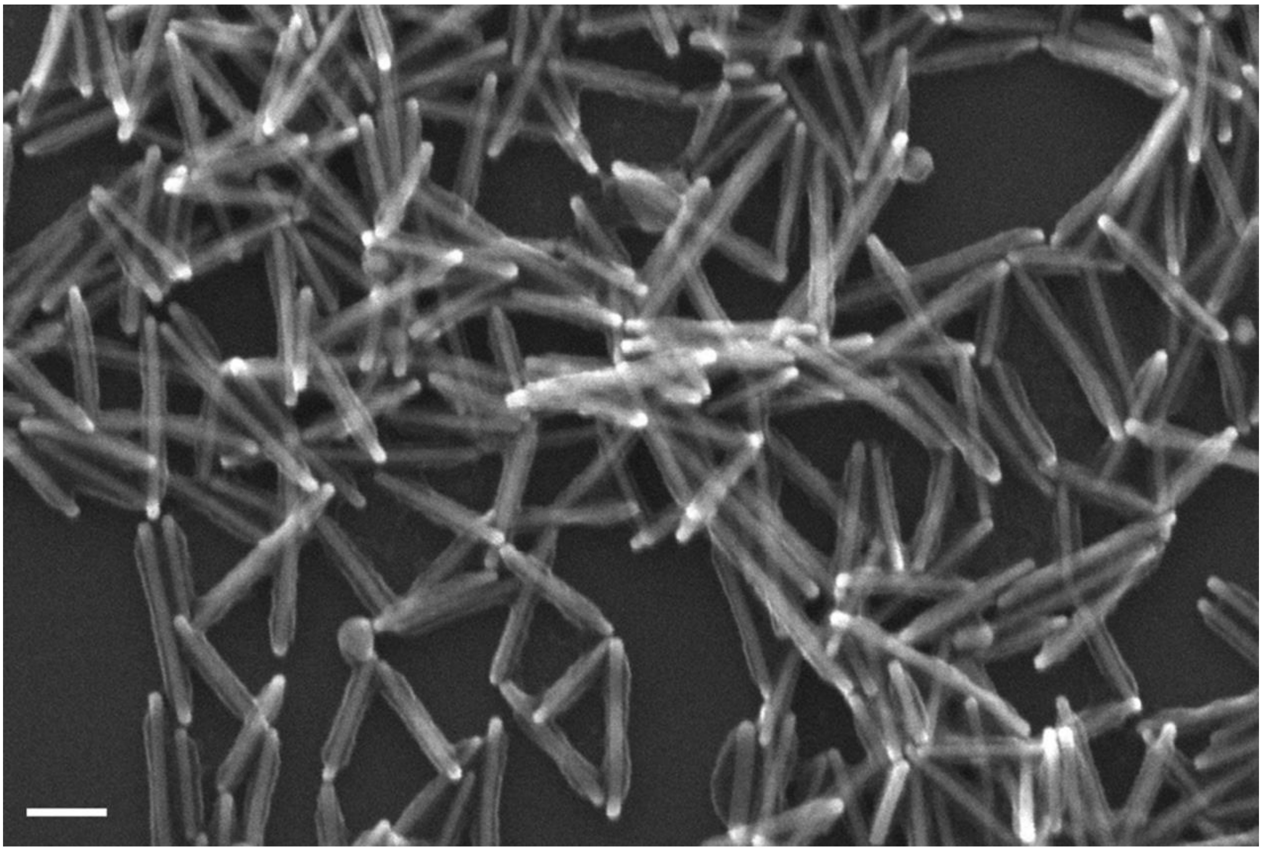

Supplementary Figure 9. SEM images of (AuNR-**1**)@PSPAA after being heated in water at 105 °C for 2.5 h. (The ligand-**1** concentration in the encapsulation step is about 0.15 mM). The scale bar is 100 nm. In the SEM data, the polymer shells can be clearly visualized, showing the exposed tips.

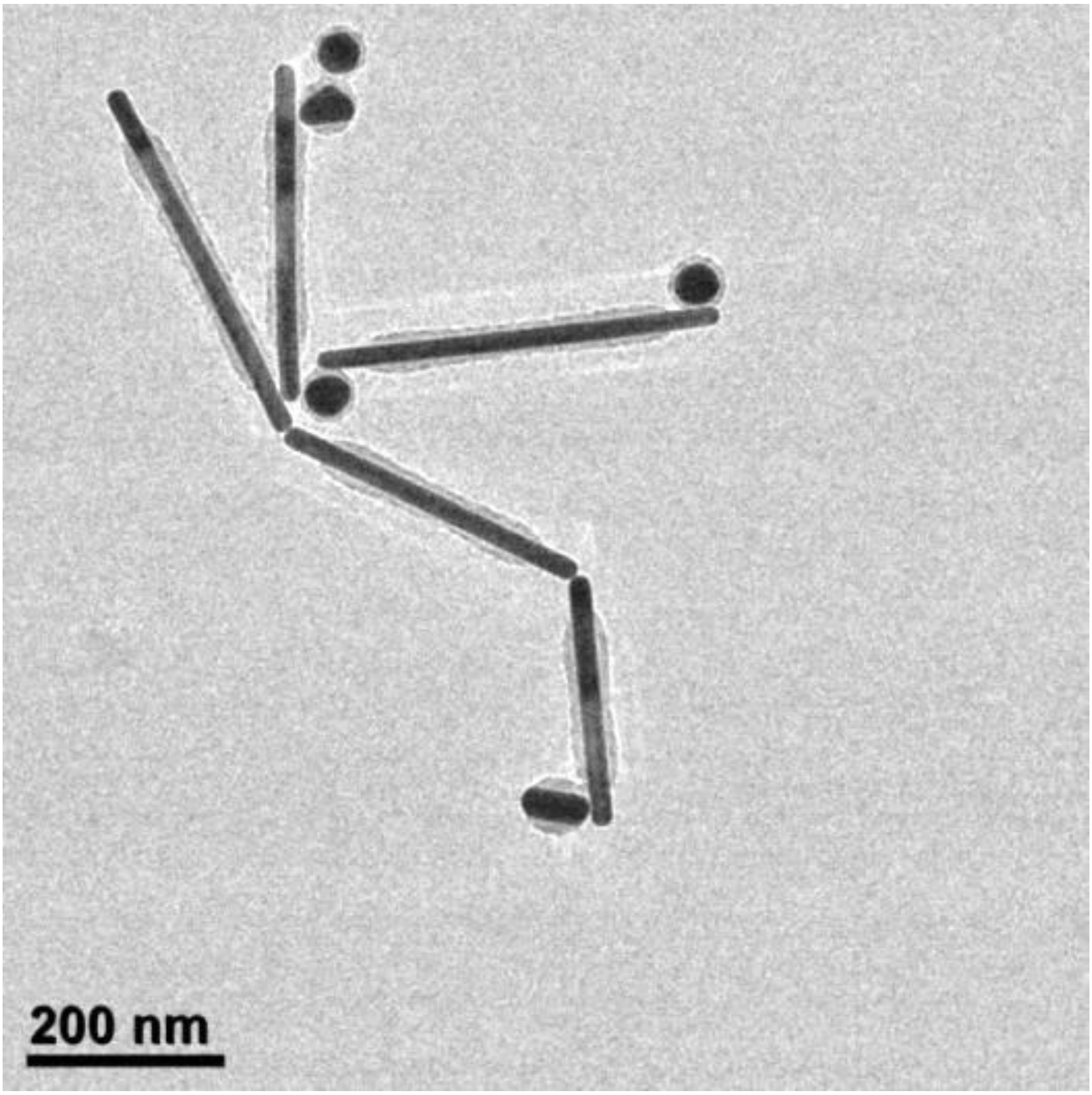

Supplementary Figure 10. TEM image of (AuNR-**1**)@PSPAA after being heated in water at 105 °C for 4 h. (The ligand-**1** concentration in the encapsulation step is about 0.15 mM).

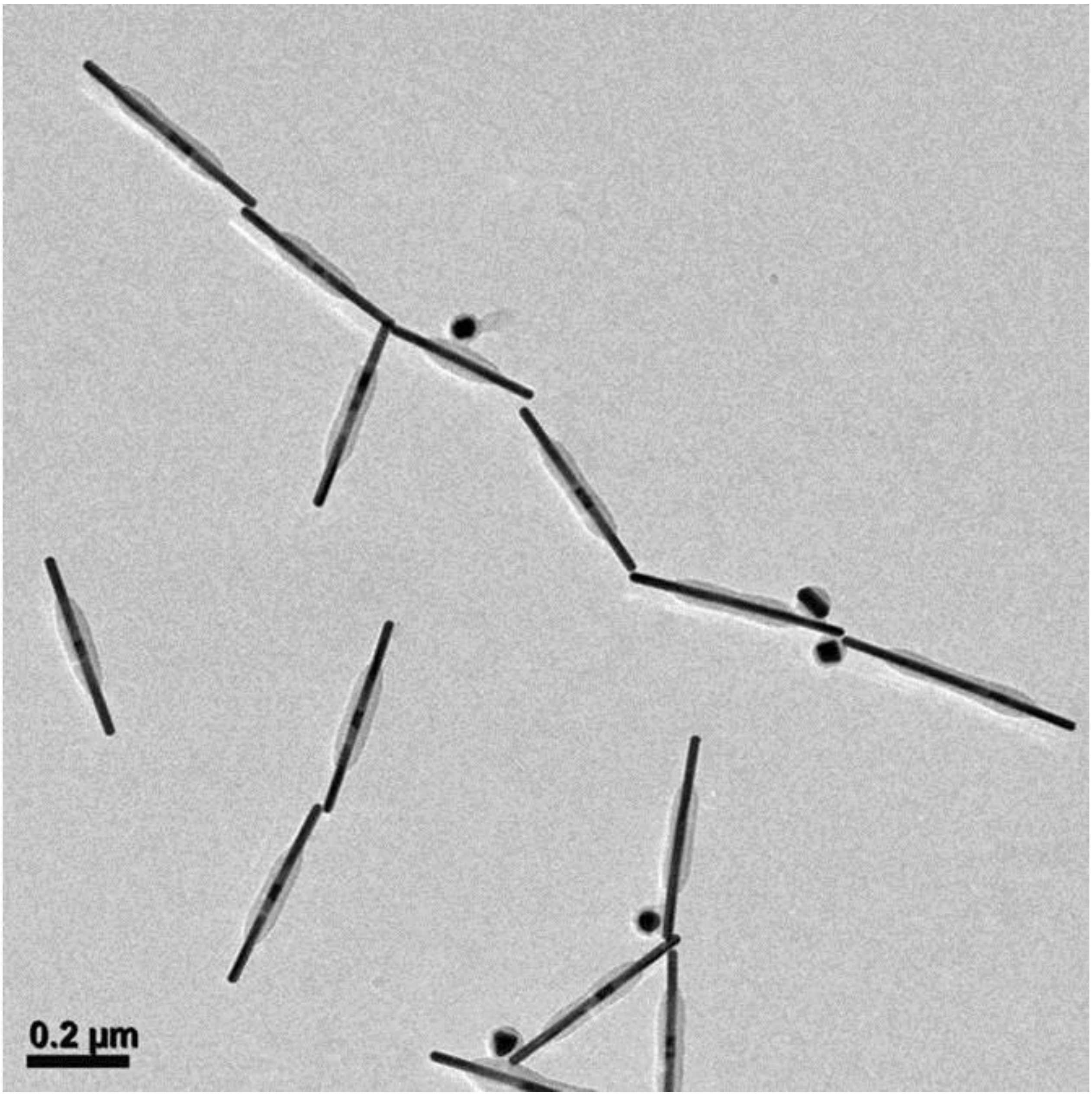

Supplementary Figure 11 TEM image of (AuNR-**1**)@PSPAA after being heated in water at 110 °C for 3 h. (The ligand-**1** concentration in the encapsulation step is about 0.15 mM).

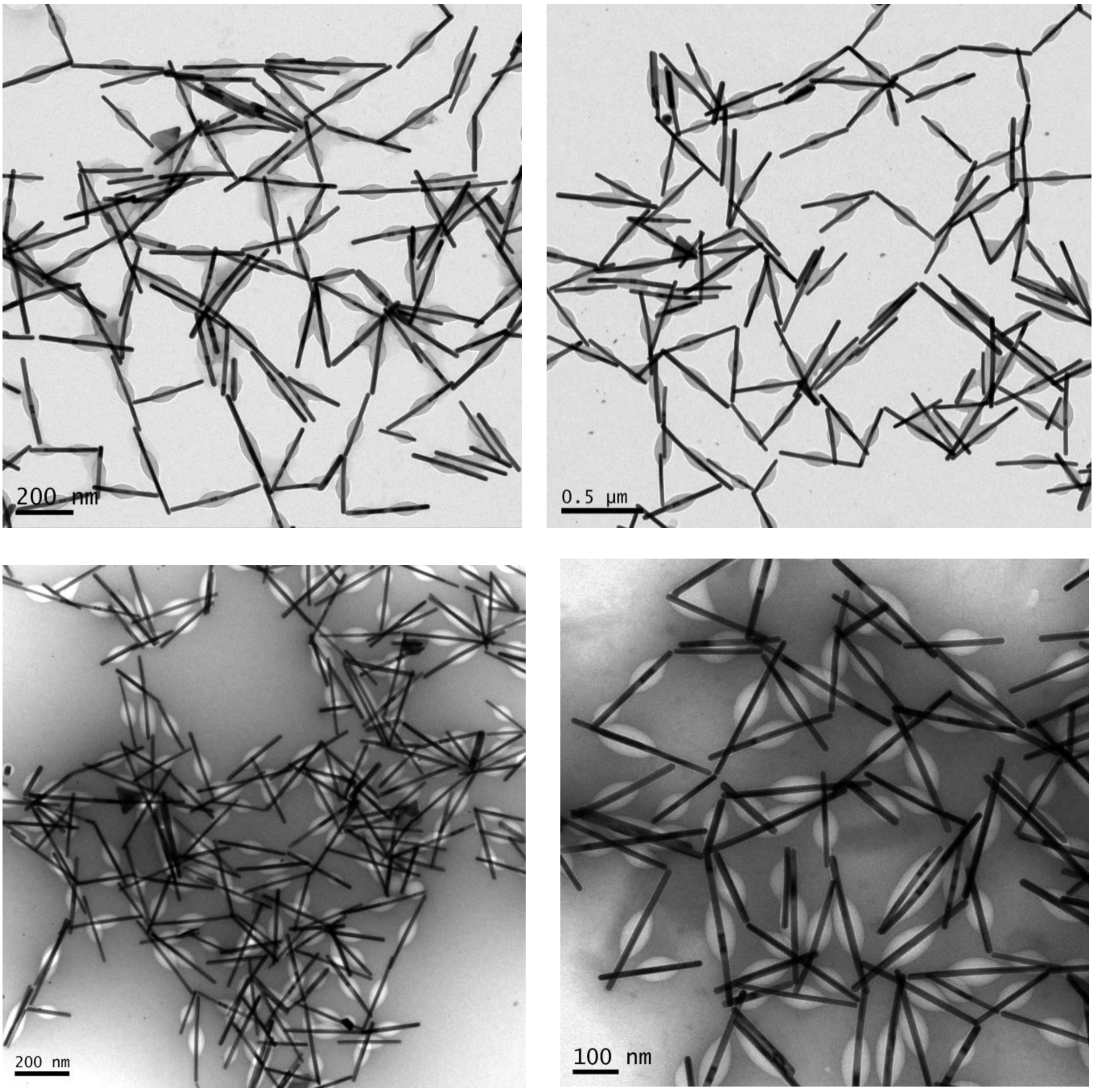

Supplementary Figure 12. TEM images of (AuNR-**1**)@PSPAA after being heated in water at 115 °C for 3 h. (The ligand-**1** concentration in the encapsulation step is about 0.15 mM).

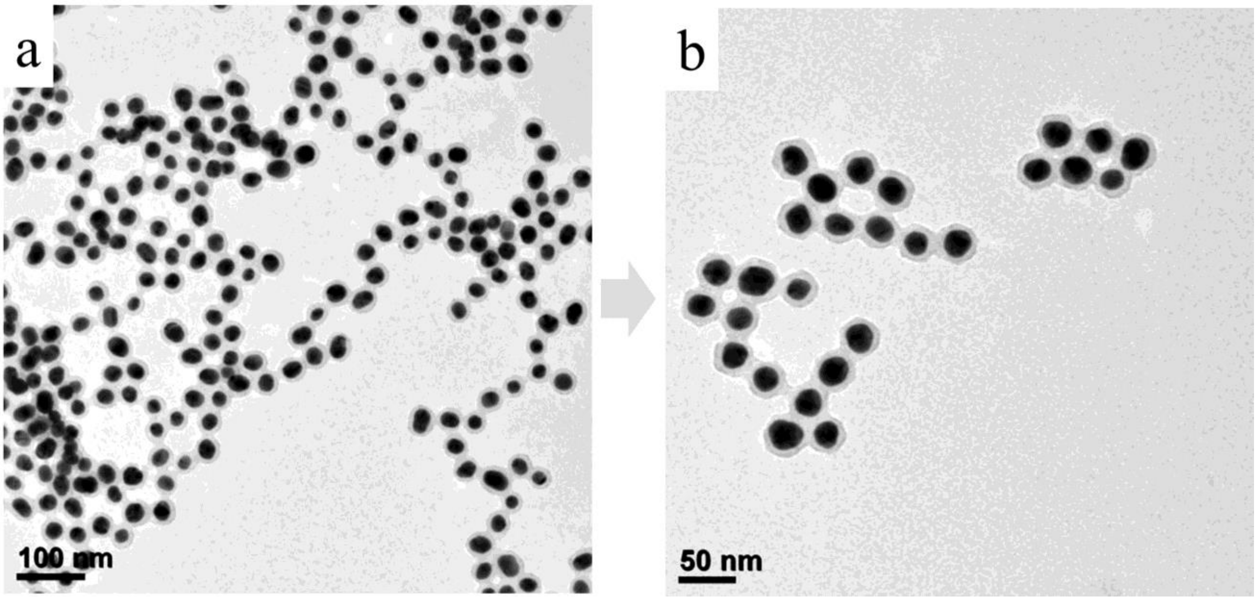

Supplementary Figure 13. TEM images of (AuNS-**1**)@PSPAA before (a) and after (b) being heated in water at 115 °C for 3 h.

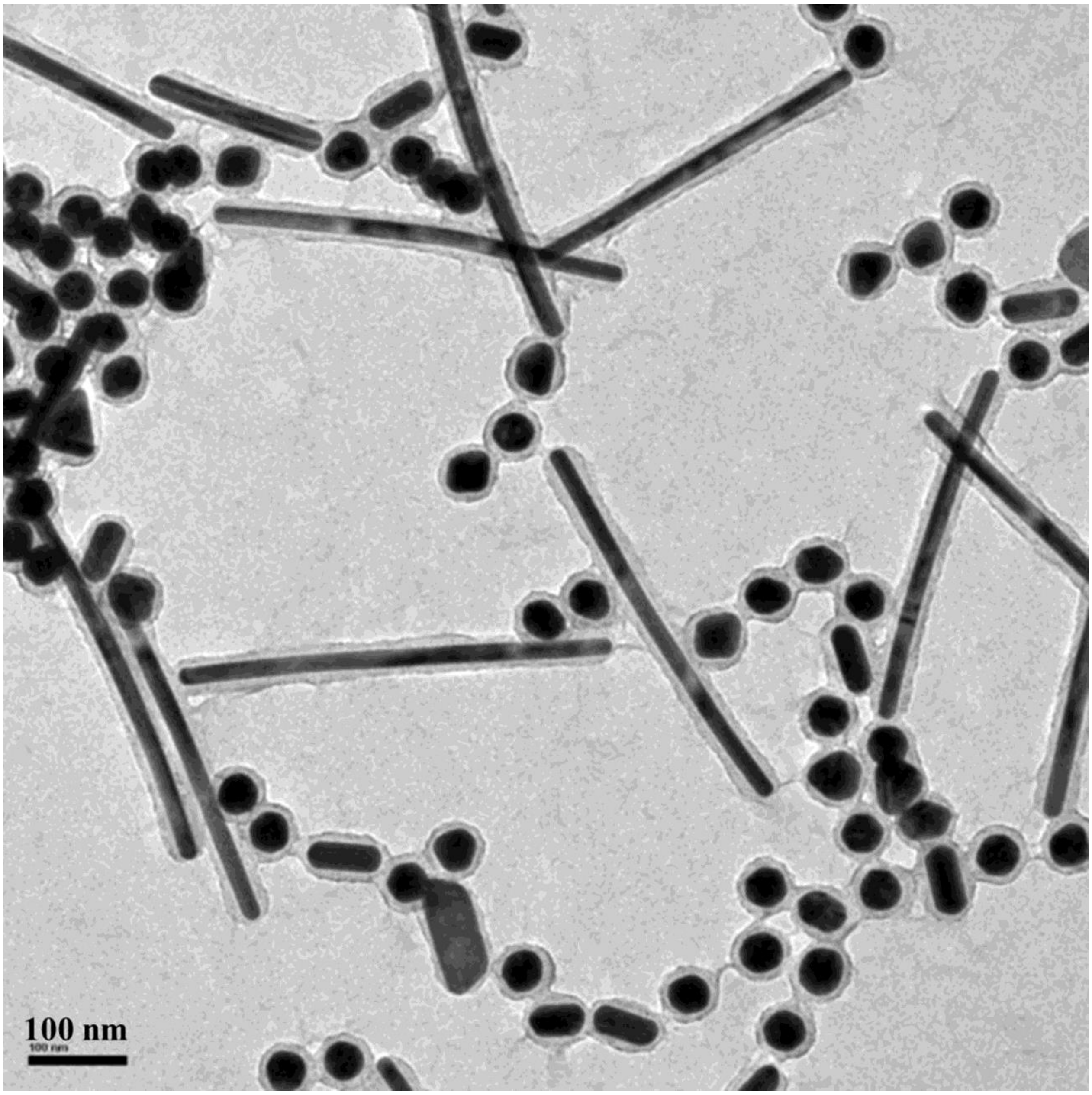

Supplementary Figure 14. TEM image of (AuNR-**2**)@PSPAA after being heated in water at 90 °C for 4 h. (The ligand-**2** concentration in the encapsulation step is about 0.27 mM). No obvious change was observed when the heating was extended to 6 h.

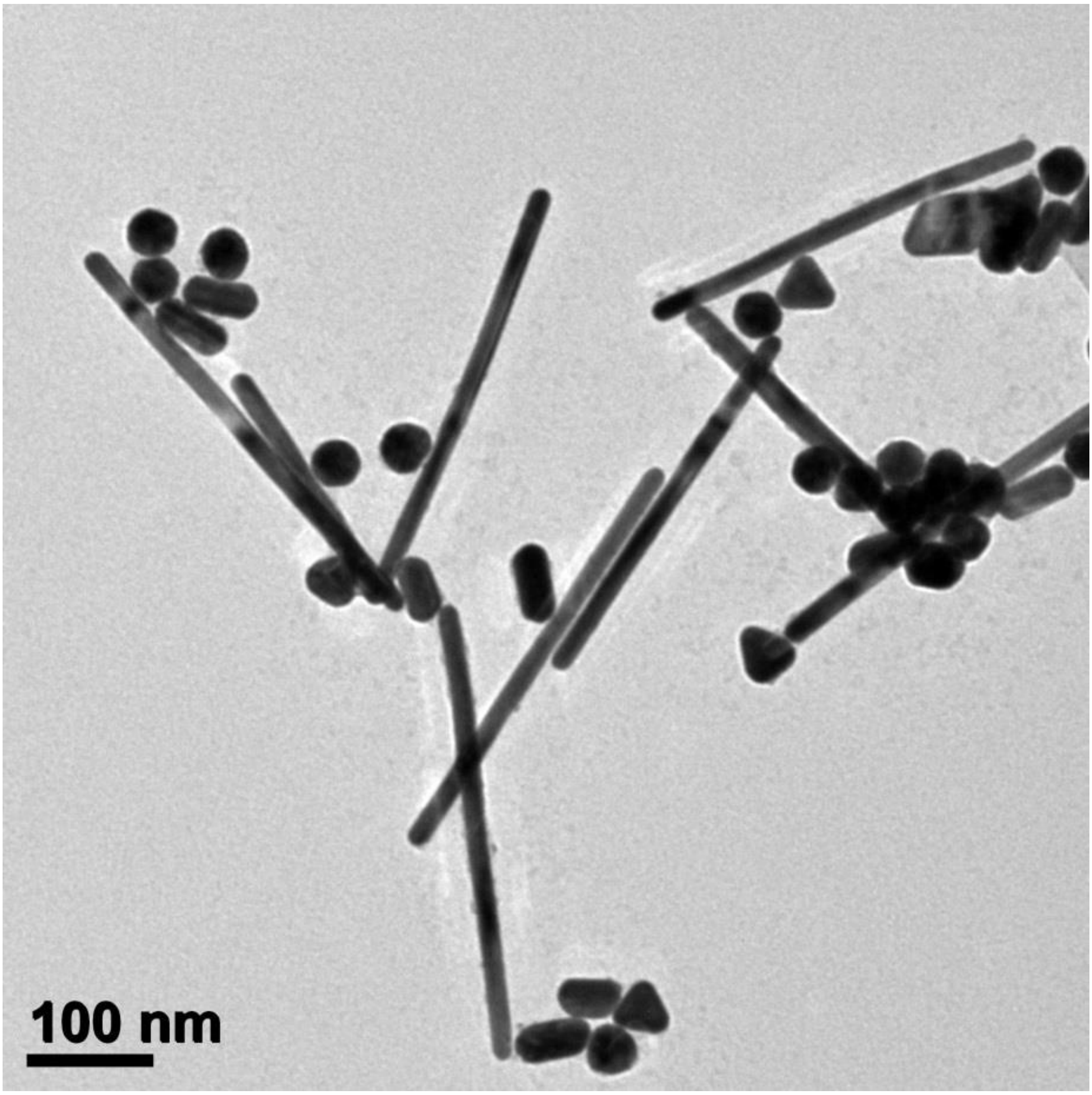

Supplementary Figure 15. TEM image of (AuNR-**3**)@PSPAA after being heated in water at 90 °C for 3 h. (The concentration of ligand-**3** in the encapsulation step is about 0.06 mM).

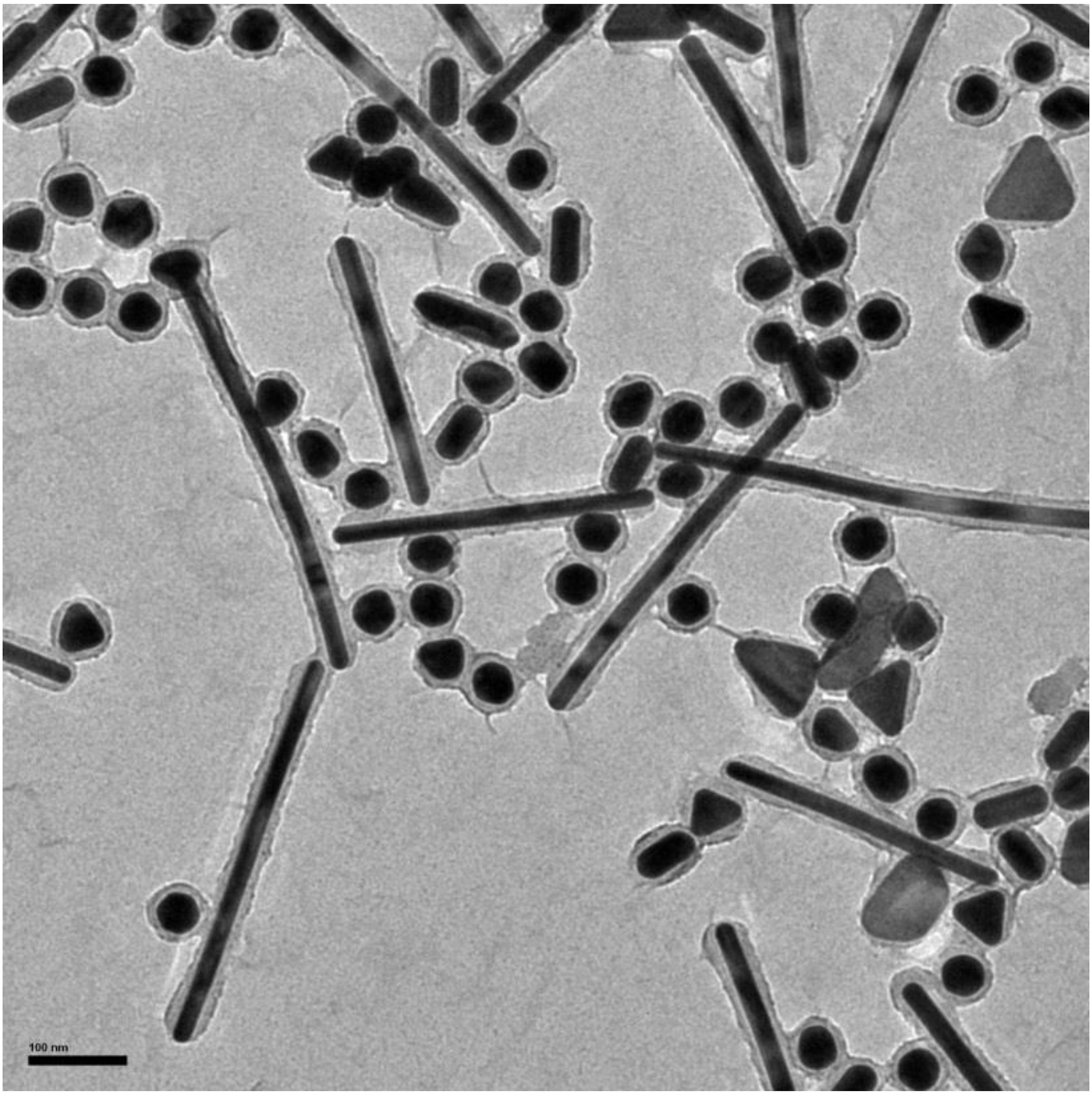

Supplementary Figure 16. TEM image of (AuNR-**3**)@PSPAA after being heated in water at 90 °C for 3 h. (The concentration of ligand-**3** in the encapsulation step is about 0.013 mM).

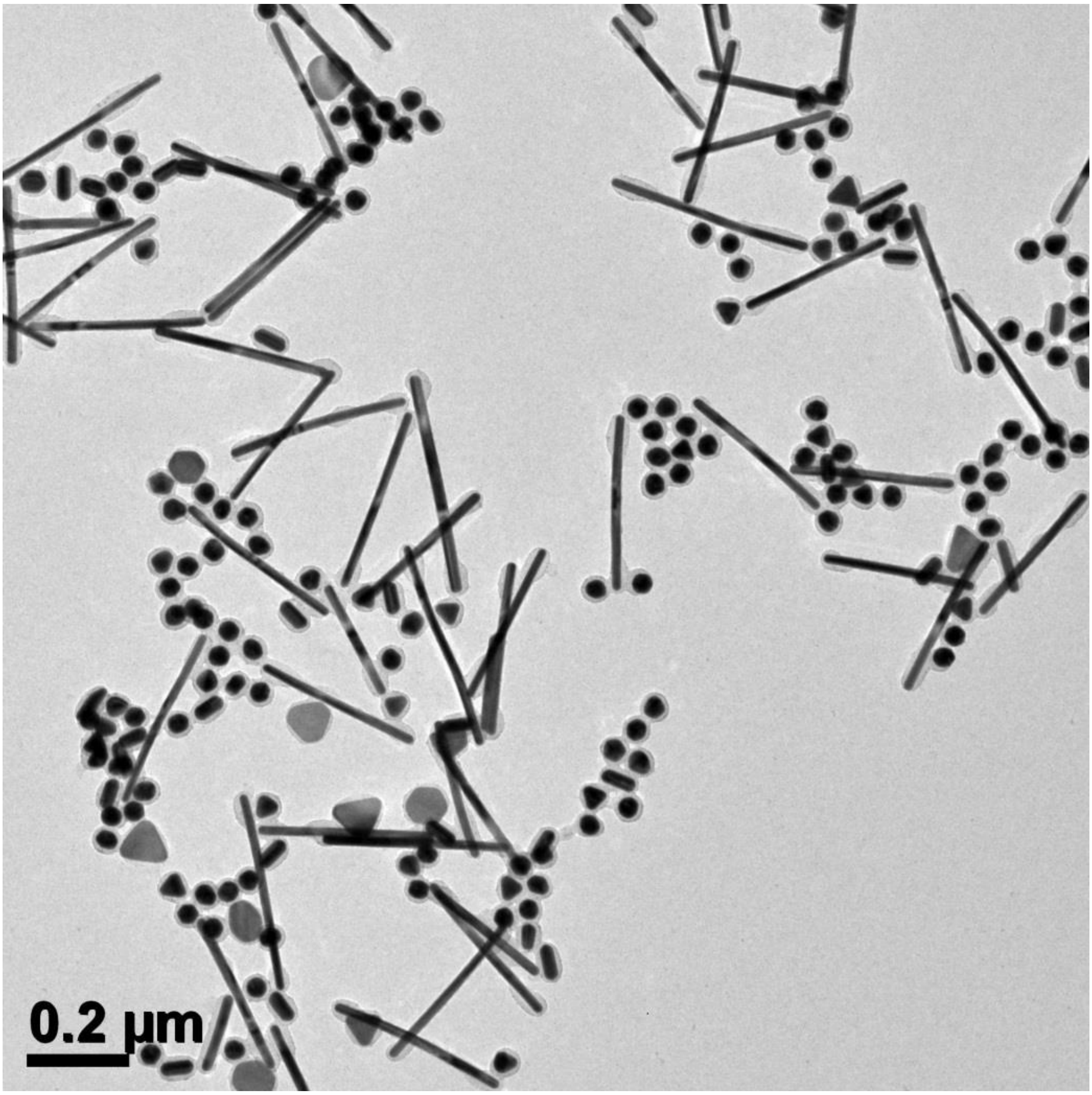

Supplementary Figure 17. TEM image of (AuNR-**3**)@PSPAA after being heated in water at 105 °C for 3 h. (The concentration of ligand-**3** in the encapsulation step is about 0.013 mM).

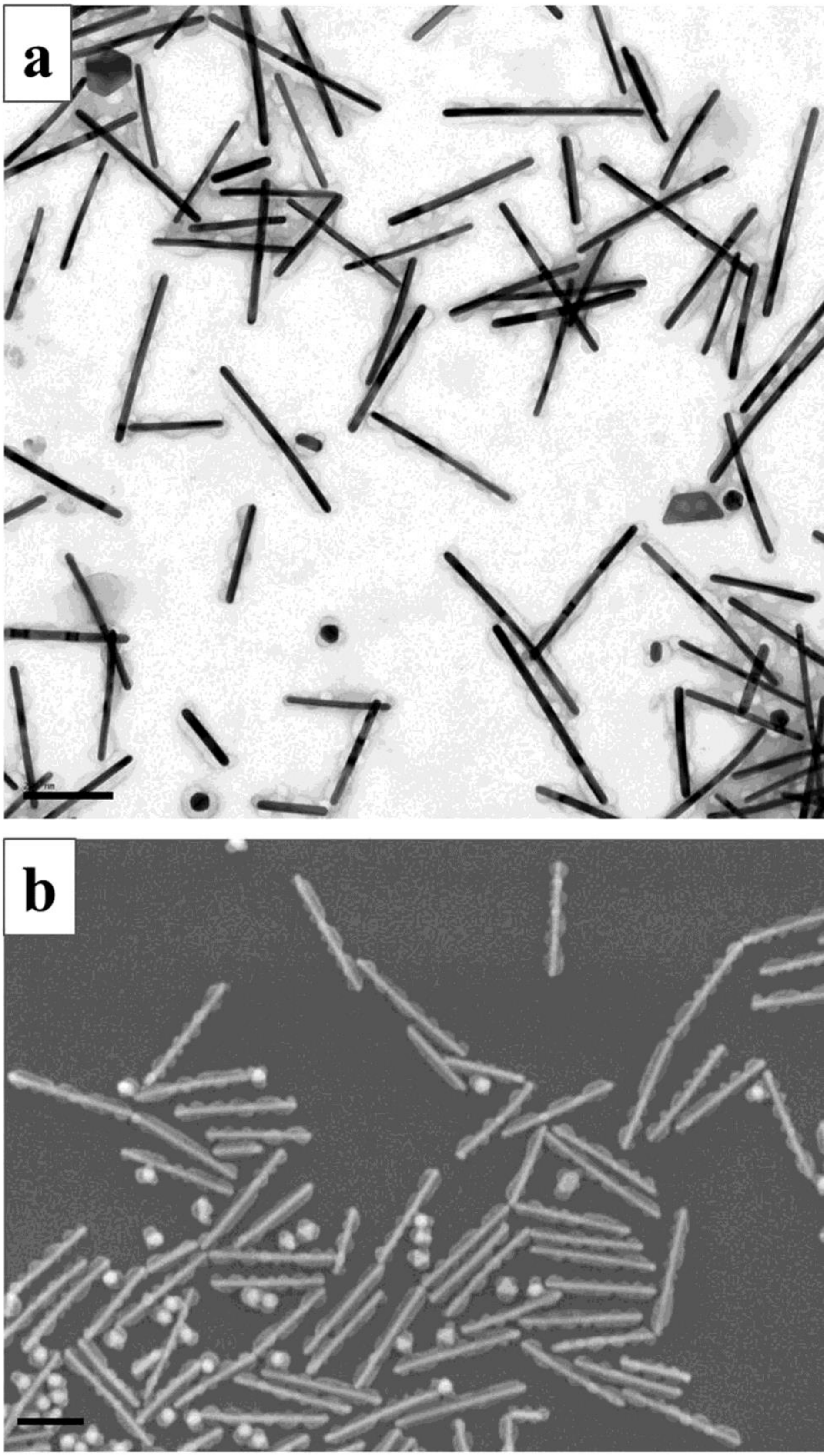

Supplementary Figure 18. TEM (a) and SEM (b) images of (AuNR-**6**)@PSPAA after being heated in water at 60 °C for 3 h. (The concentration of ligand-**6** in the encapsulation step is about 0.12 mM).The scale bars are 200 nm.

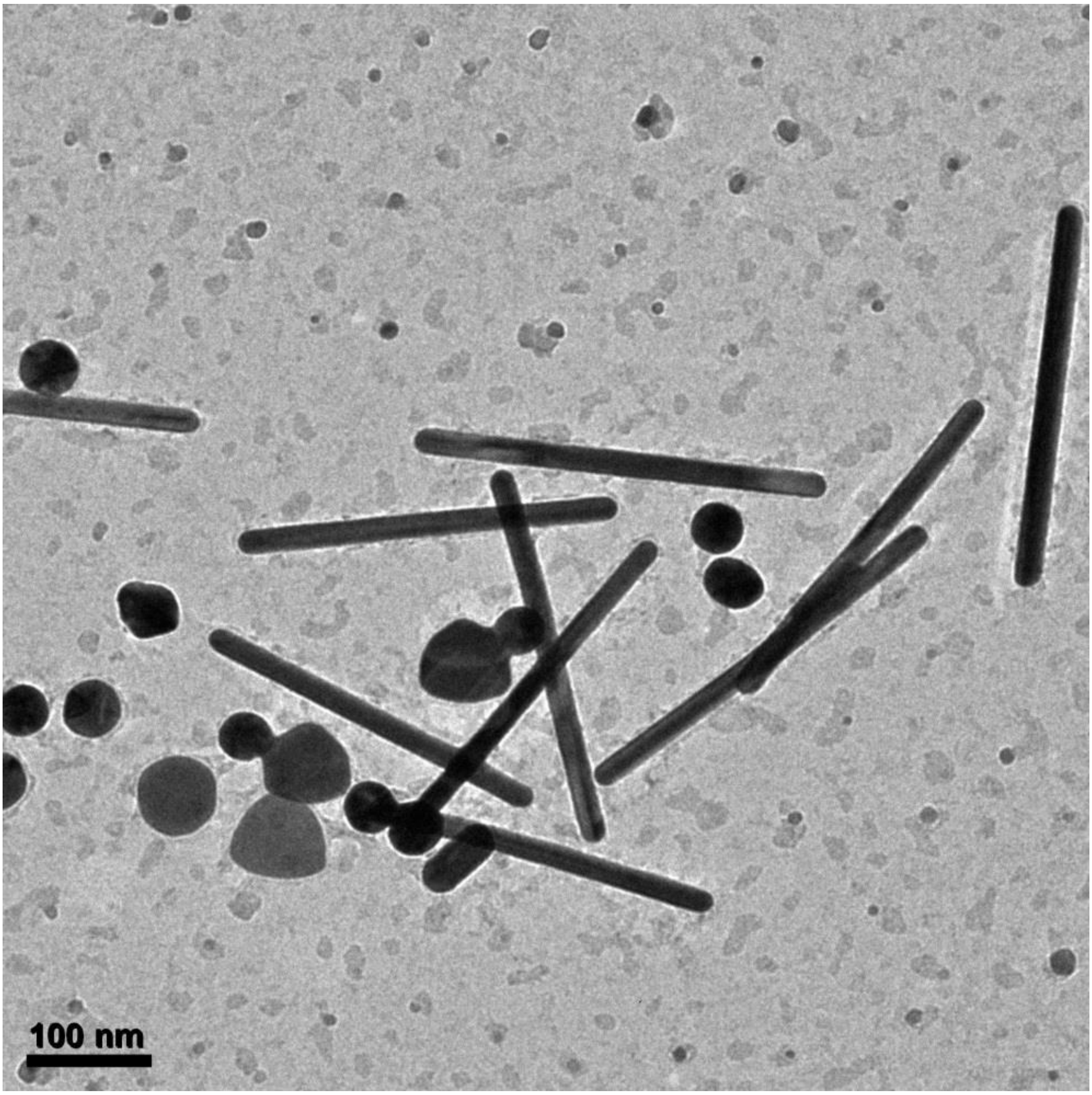

Supplementary Figure 19. TEM image of (AuNR-**6**)@PSPAA after being heated in water at 60 °C for 3 h. (The concentration of ligand-**6** in the encapsulation step is about 0.20 mM).

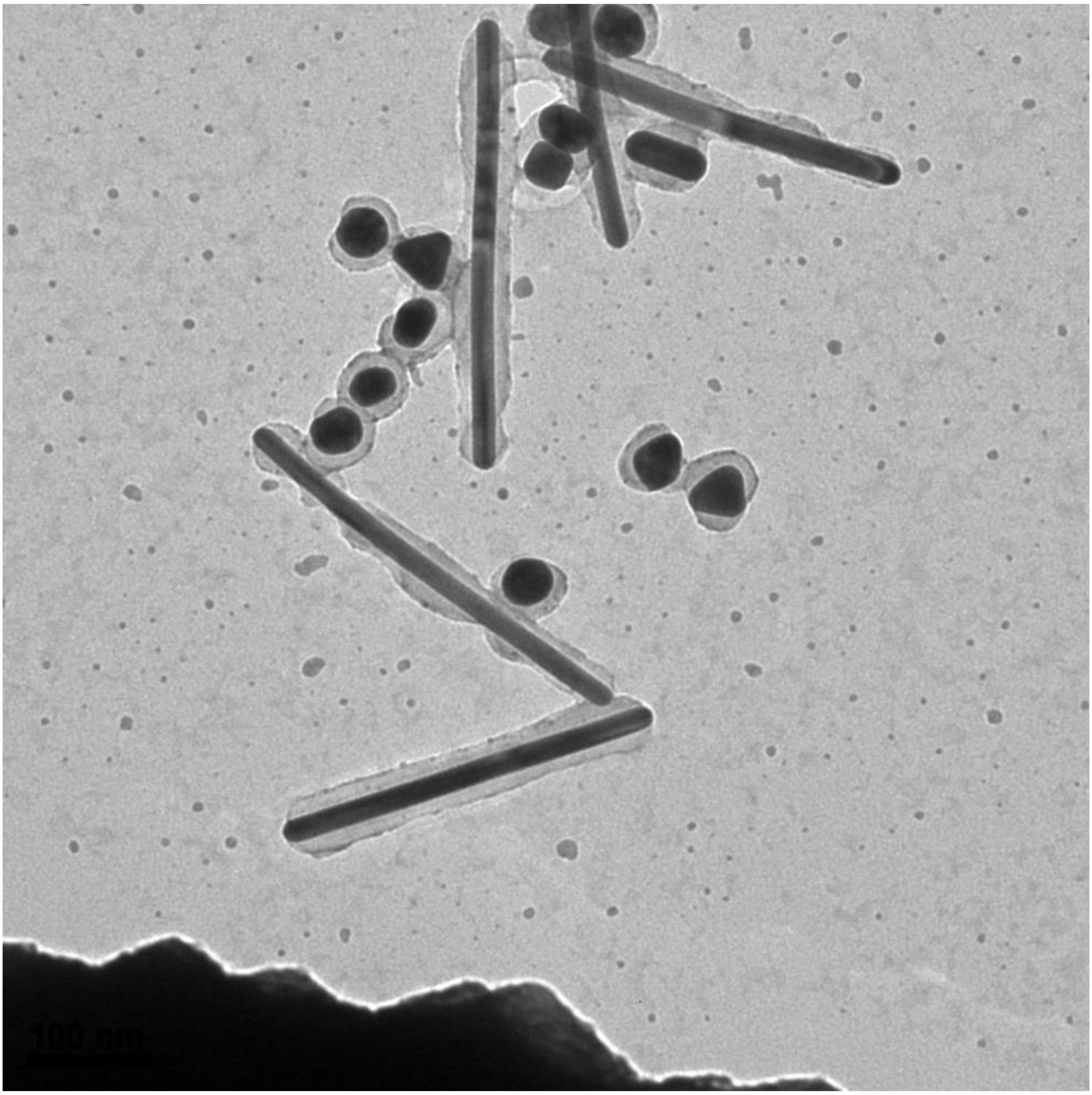

Supplementary Figure 20. TEM image of (AuNR-**6**)@PSPAA after being heated in water at 60 °C for 3 h. (The concentration of ligand-**6** in the encapsulation step is about 0.08 mM).

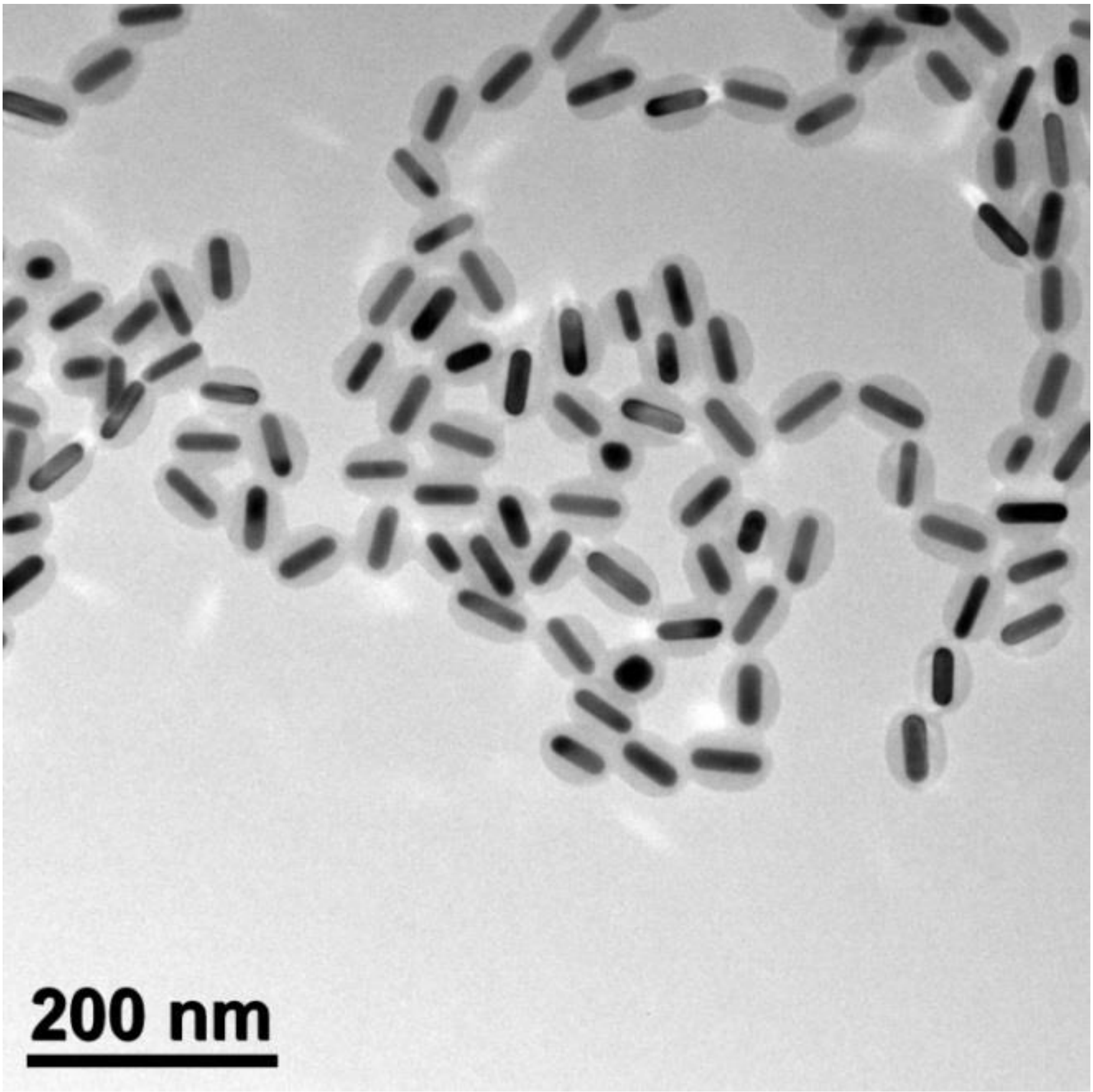

Supplementary Figure 21. TEM image of the pristine core-shell structure of (AuNR-**6**)@PSPAA (before transformation).

Ligand	C (mM)[a]	T (°C)[b]	Time (h)	Transformation Mode
1 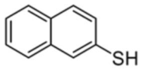	0.10	90	4	
	1.50	90	4	
	0.15	90	1	
	0.15	90	3	
	0.15	90	5	
	0.15	90	12	
	0.15	105	1.5	
	0.15	105	2.5	
	0.15	105	4	
	0.15	105	6	
	0.15	110	3	
	0.15	110	5	
	0.15	115	3	
	0.15	115	5	

Ligand	C (mM)	T (°C)	Time (h)	Transformation Mode
2 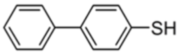	0.27	90	4	
	1.60	90	3	
	1.60	90	4	
	1.60	90	5	
	1.60	90	6	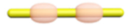
3 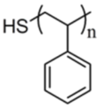	0.013	90	3	
	0.026	90	1	
	0.026	90	2	
	0.026	90	3	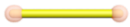
	0.013	110	3	
	0.060	90	3	
4 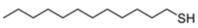	0.10	90	3	
	0.20	90	3	
5	0.10	90	3	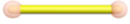
	0.20	90	3	
6 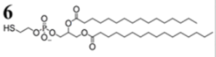	0.08	60	3	
	0.12	60	3	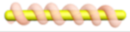
	0.20	60	3	

Supplementary Figure 22. The detailed reaction conditions leading to the four transformation modes. [a] the concentration of ligand in the initial encapsulation step; [b] the temperature for heating the water dispersed (AuNR-**ligand**)@PSPAA.

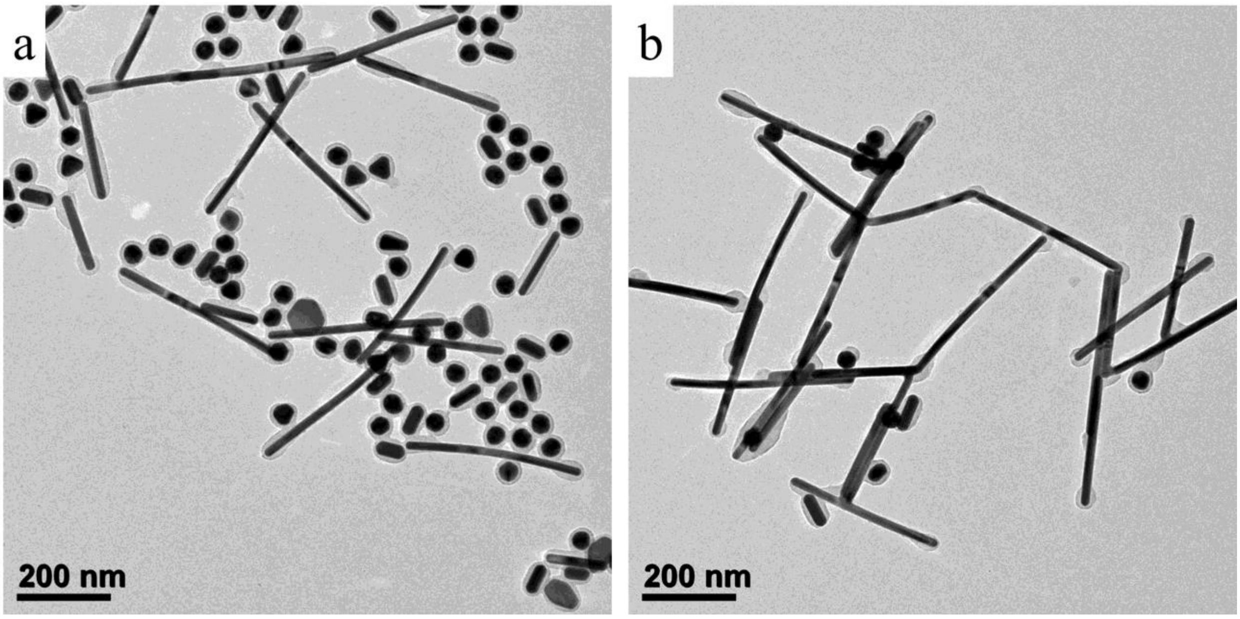

Supplementary Figure 23. TEM images of (AuNR-**ligand**)@PSPAA after being heated in water at 90 °C for 3 h. (a) The ligand used in the encapsulation step is 1-dodecanethiol (ligand-**4**), and the concentration of the ligand is about 0.1 mM)**;** (b) The ligand used in the encapsulation step is 1-octadecanethiol (ligand-**5**), and the concentration of the ligand is about 0.1 mM.

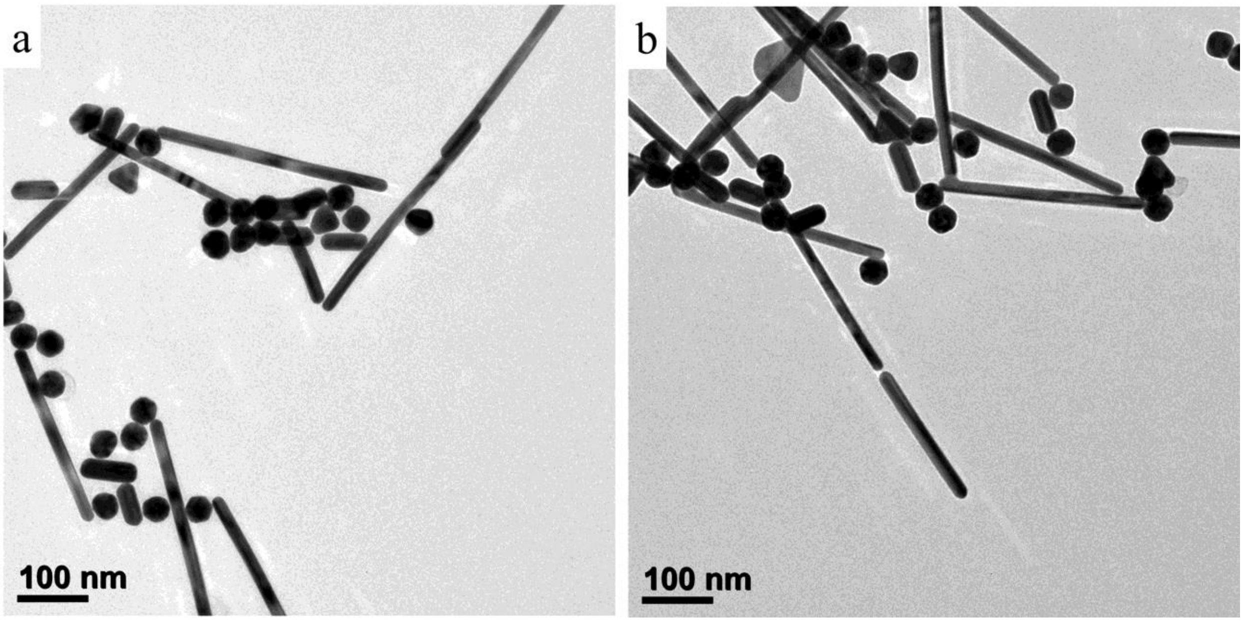

Supplementary Figure 24. TEM images of (AuNR-**ligand**)@PSPAA after being heated in water at 90 °C for 3 h. (a) The ligand used in the encapsulation step is 1-dodecanethiol (ligand-**4**), and the concentration of the ligand is about 0.2 mM)**;** (b) The ligand used in the encapsulation step is 1-octadecanethiol (ligand-**5**), and the concentration of the ligand is about 0.2 mM.

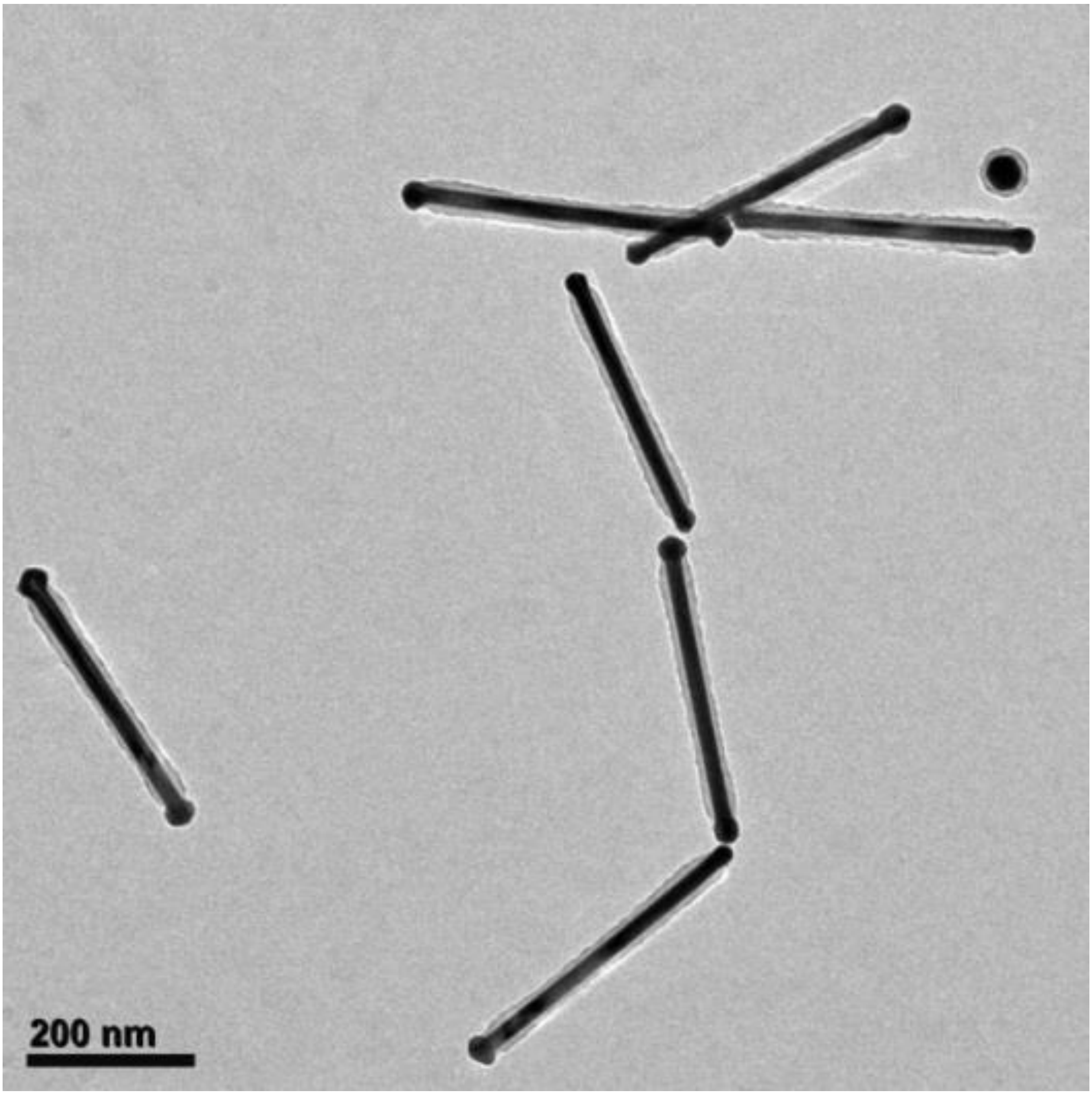

Supplementary Figure 25. TEM image of silver deposition on the transformed (AuNR-**1**)@PSPAA. The transformed (AuNR-**1**)@PSPAA are prepared by heating in water at 105 °C for 1.5 h. (The ligand-**1** concentration in the encapsulation step is about 0.15 mM).

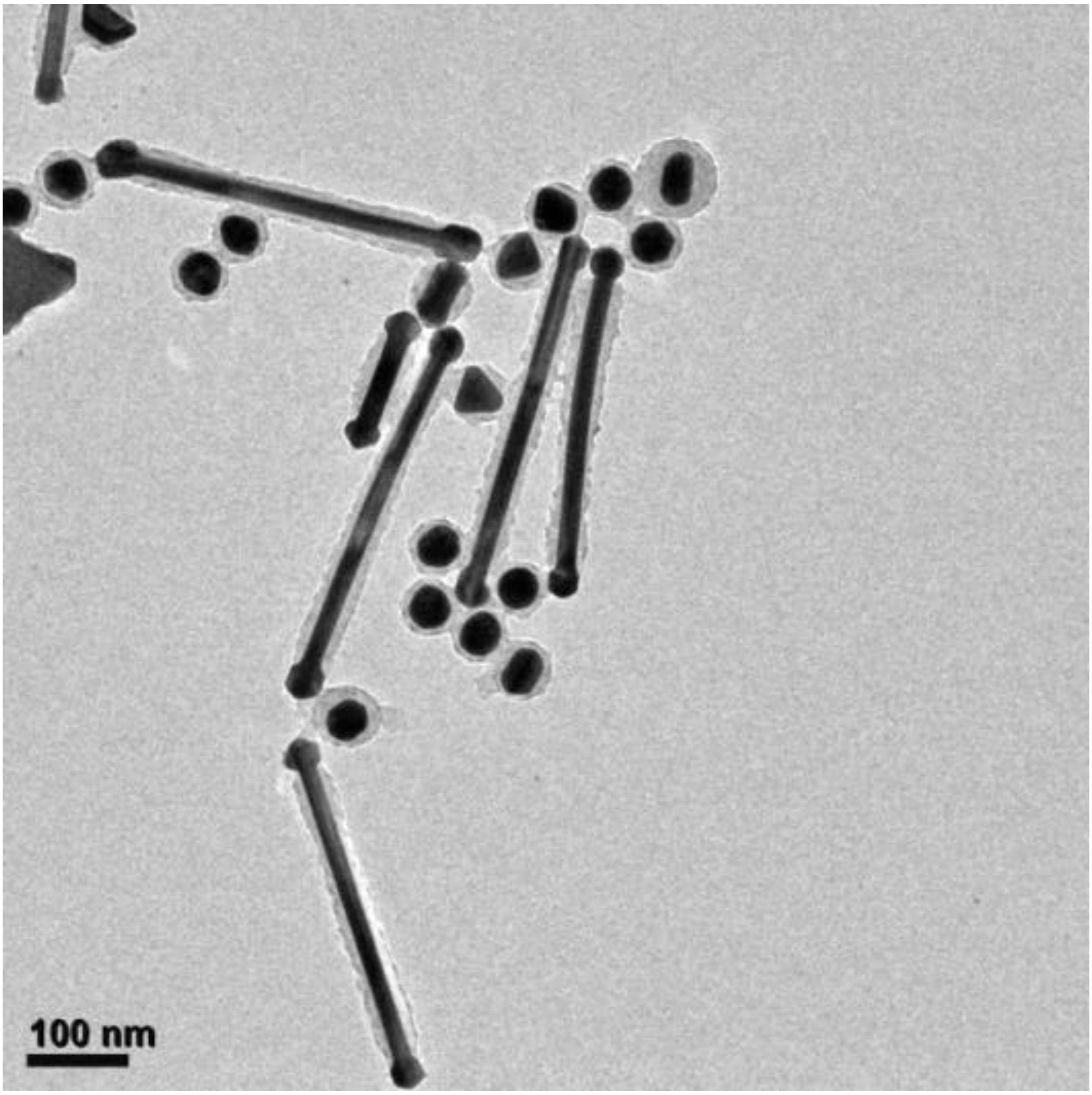

Supplementary Figure 26. TEM image of silver deposition on the transformed (AuNR-**1**)@PSPAA. The transformed (AuNR-**1**)@PSPAA are prepared by heating in water at 105 °C for 2.5 h. (The ligand-**1** concentration in the encapsulation step is about 0.15 mM).

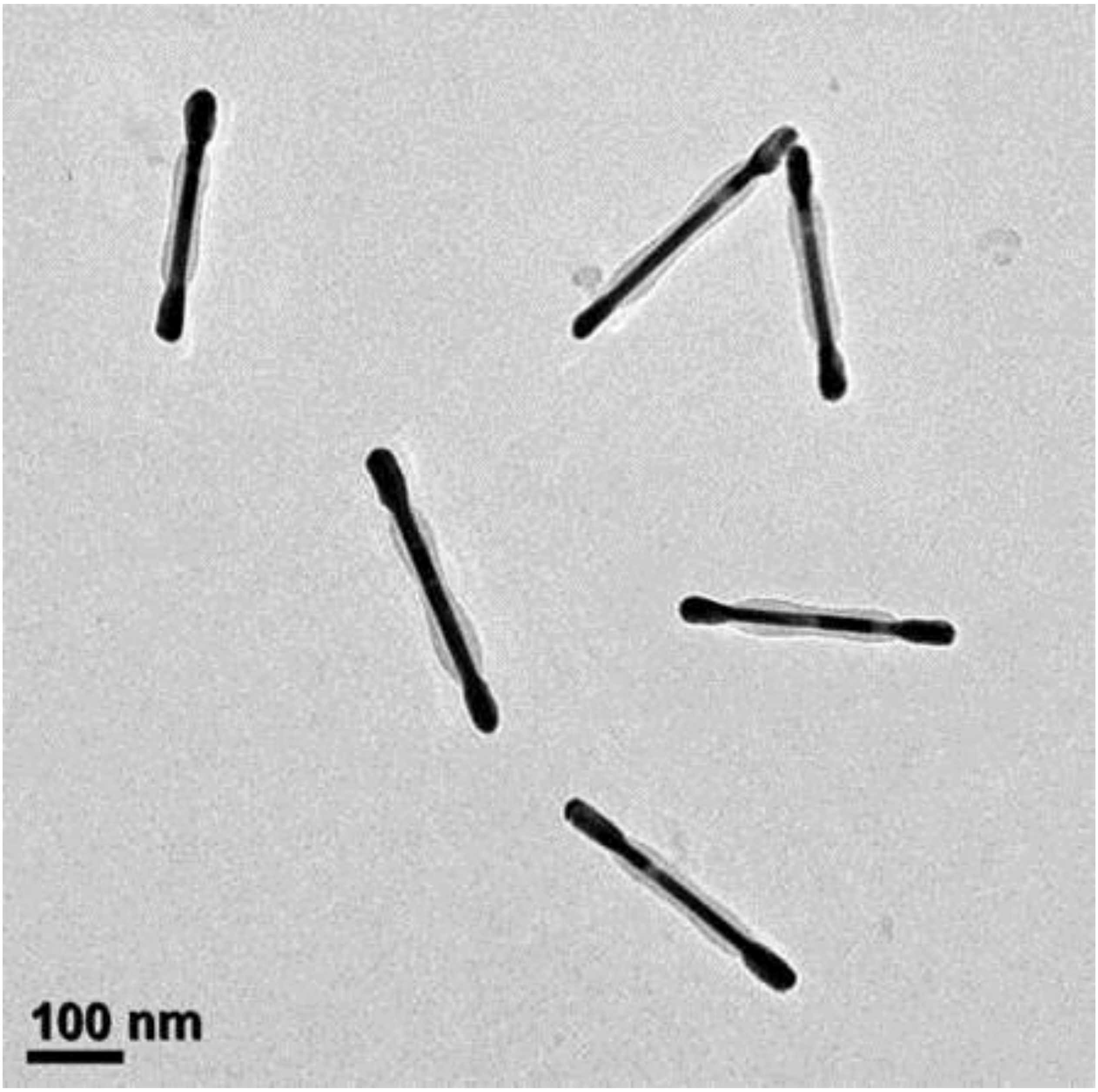

Supplementary Figure 27. TEM image of silver deposition on the transformed (AuNR-**1**)@PSPAA. The transformed (AuNR-**1**)@PSPAA are prepared by heating in water at 105 °C for 4 h. (The ligand-**1** concentration in the encapsulation step is about 0.15 mM).

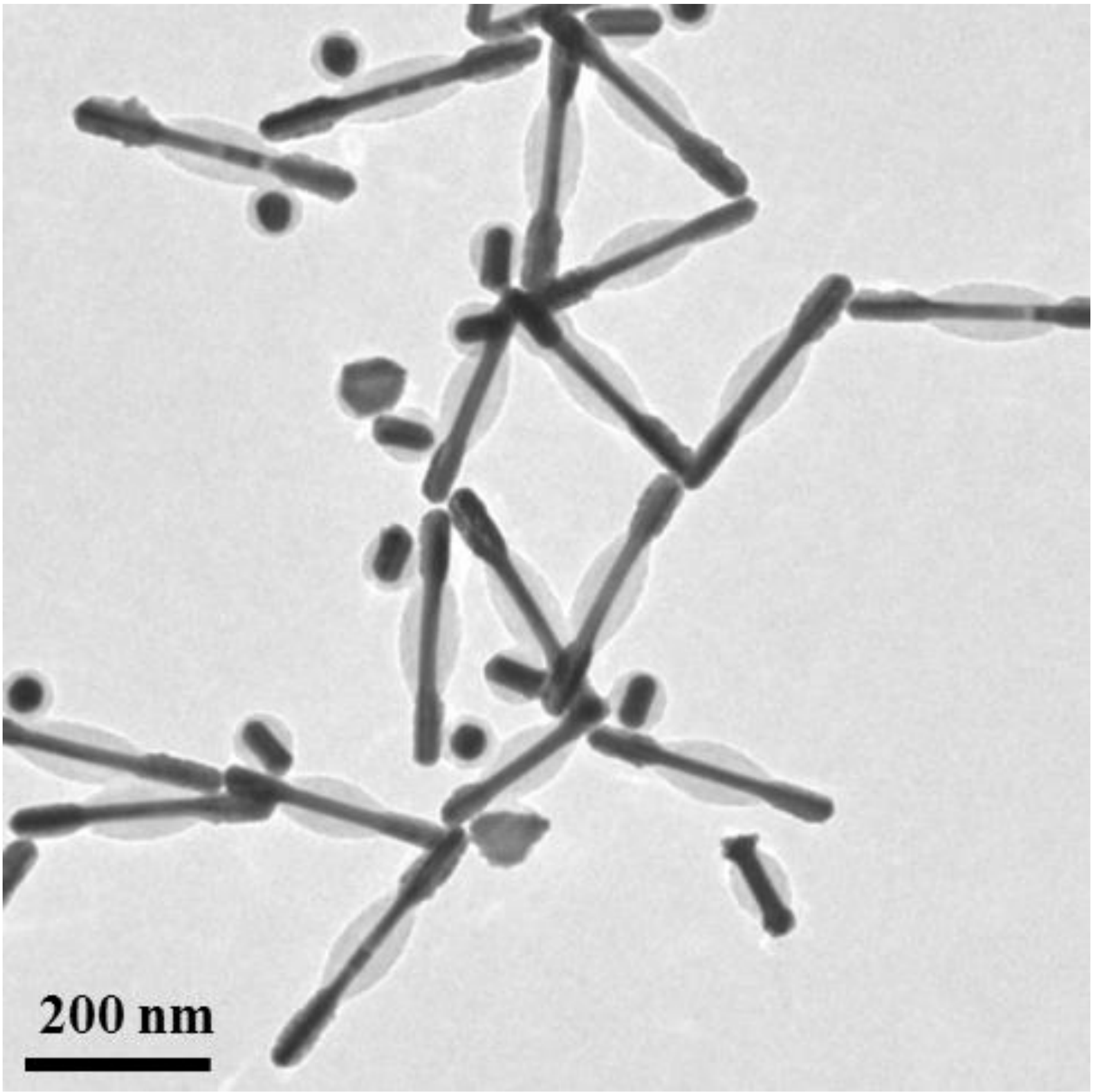

Supplementary Figure 28. TEM image of silver deposition on the transformed (AuNR-**1**)@PSPAA. The transformed (AuNR-**1**)@PSPAA are prepared by heating in water at 110 °C for 3 h. (The ligand-**1** concentration in the encapsulation step is about 0.15 mM).

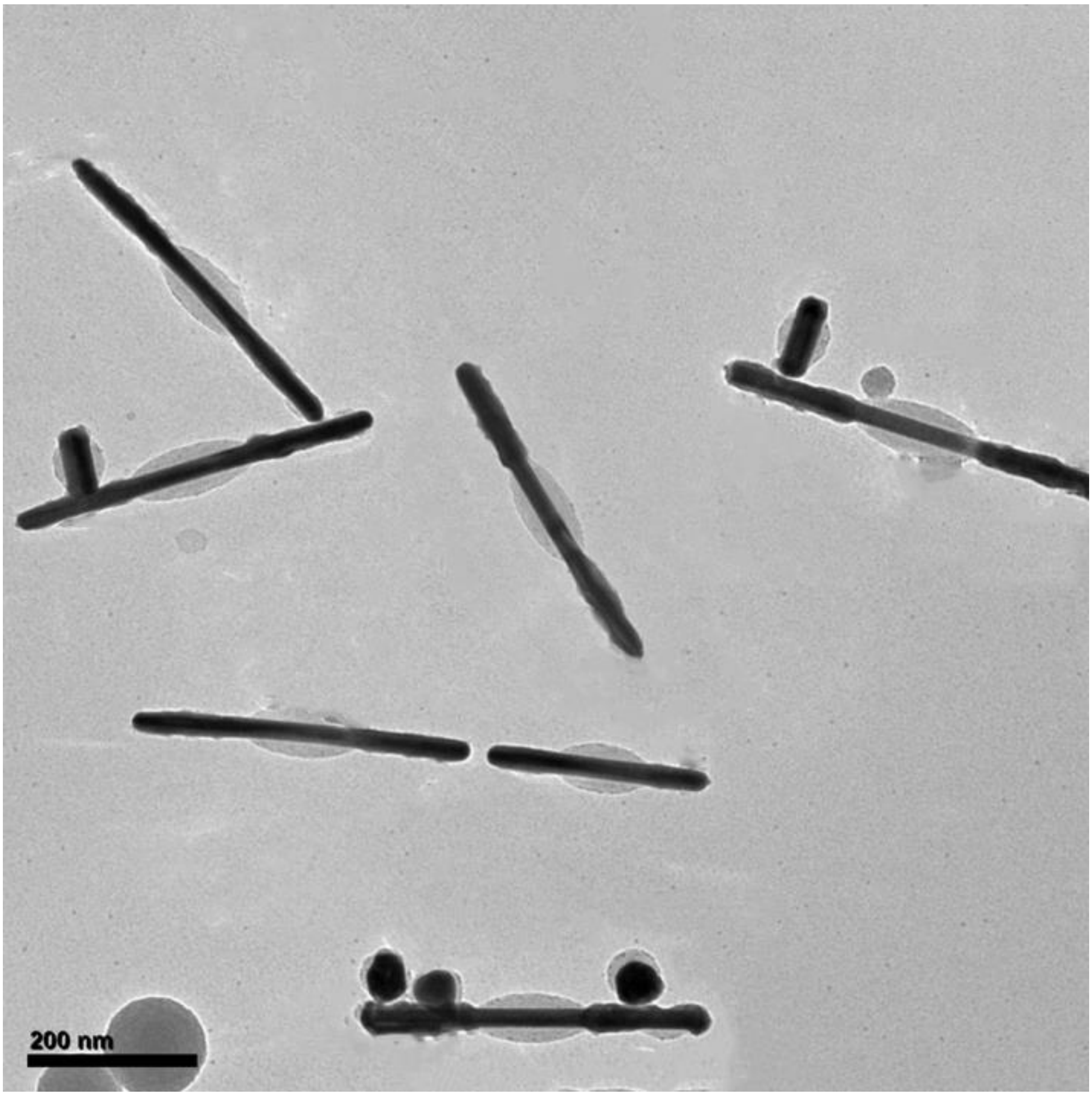

Supplementary Figure 29. TEM image of silver deposition on the transformed (AuNR-**1**)@PSPAA. The transformed (AuNR-**1**)@PSPAA are prepared by heating in water at 115 °C for 3 h. (The ligand-**1** concentration in the encapsulation step is about 0.15 mM).

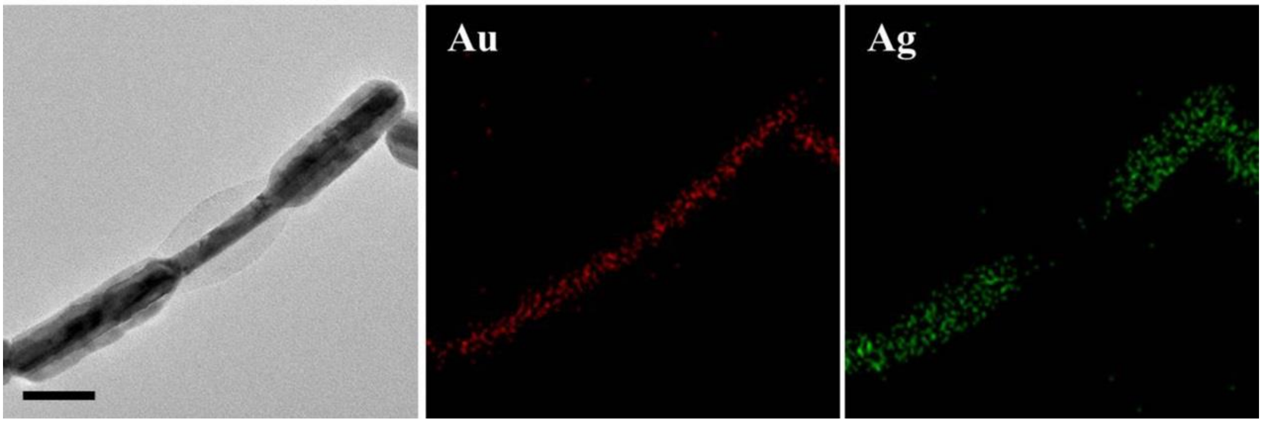

Supplementary Figure 30. TEM image and EDX mapping of the transformed (AuNR-**1**)@PSPAA with Ag tips. The transformed (AuNR-**1**)@PSPAA are prepared by heating in water at 115 °C for 3 h. (The ligand-**1** concentration in the encapsulation step is about 0.15 mM). The scale bar is 50 nm.

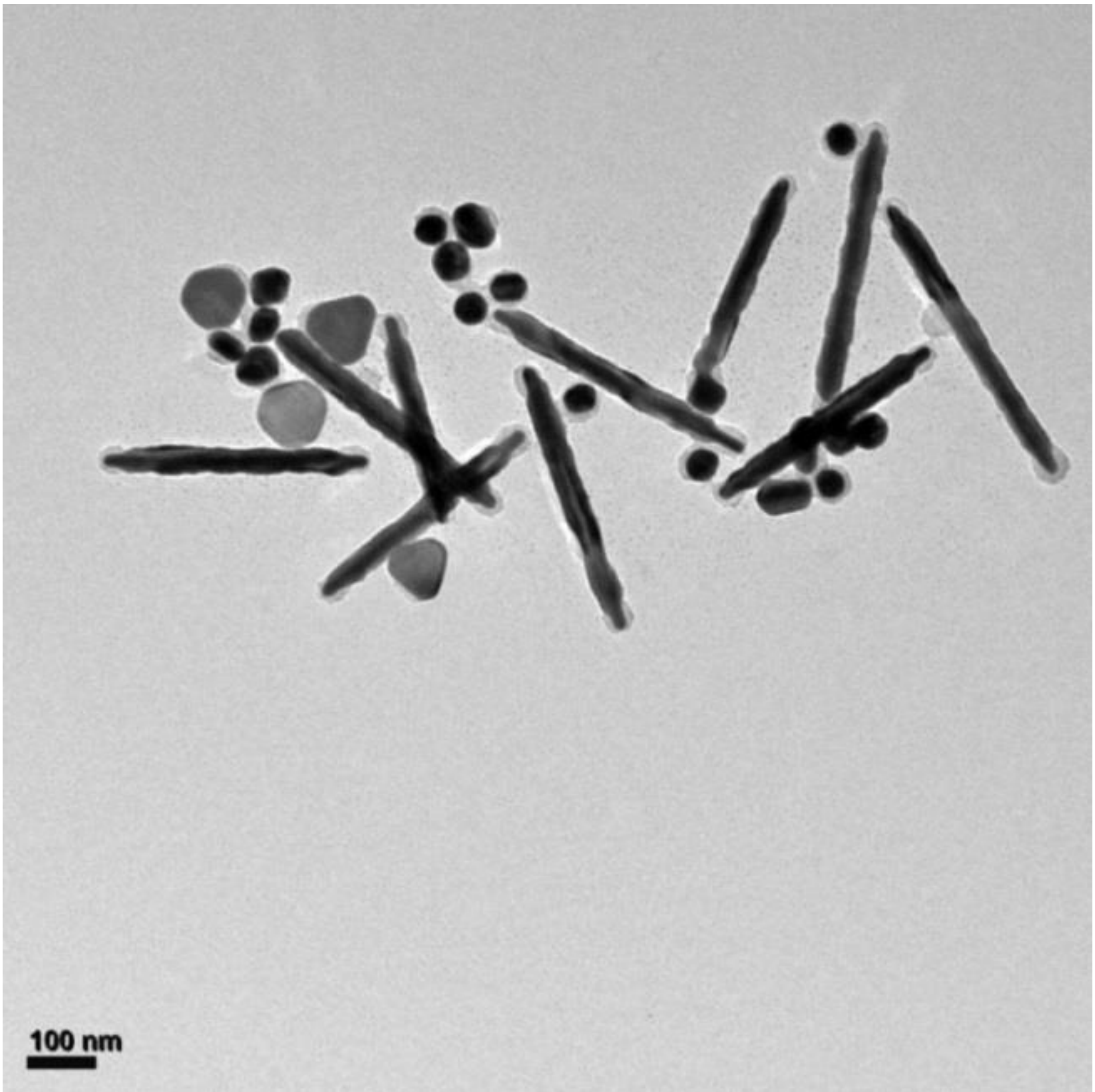

Supplementary Figure 31. TEM image of silver deposition on the transformed (AuNR-**3**)@PSPAA. The transformed (AuNR-**3**)@PSPAA are prepared by heating in water at 90 °C for 3 h. (The ligand-**3** concentration in the encapsulation step is about 0.026 mM).

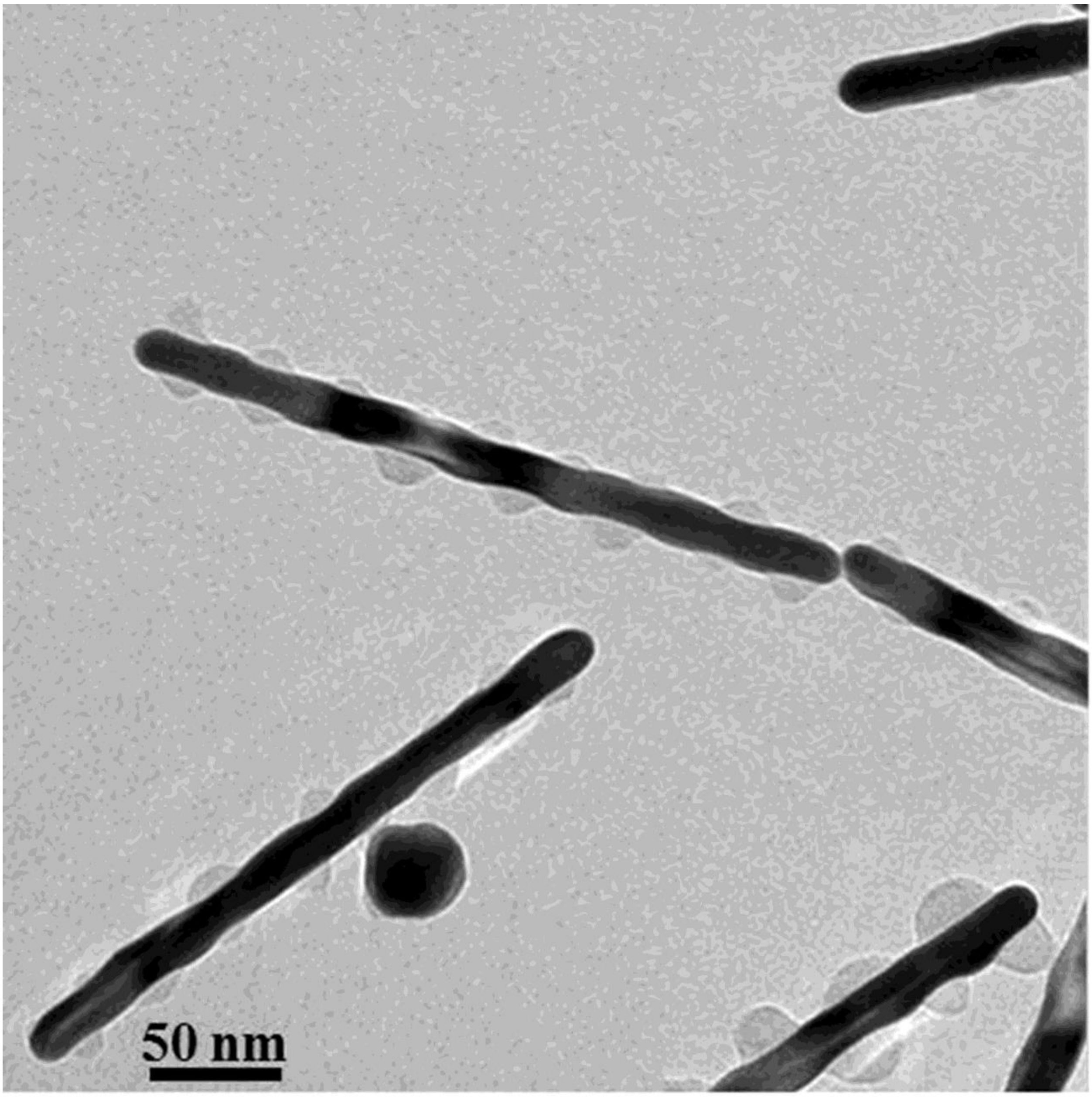

Supplementary Figure 32. TEM image of silver deposition on the transformed (AuNR-**6**)@PSPAA. The transformed (AuNR-**6**)@PSPAA are prepared by heating in water at 60 °C for 3 h. (The ligand-**6** concentration in the encapsulation step is about 0.12 mM).

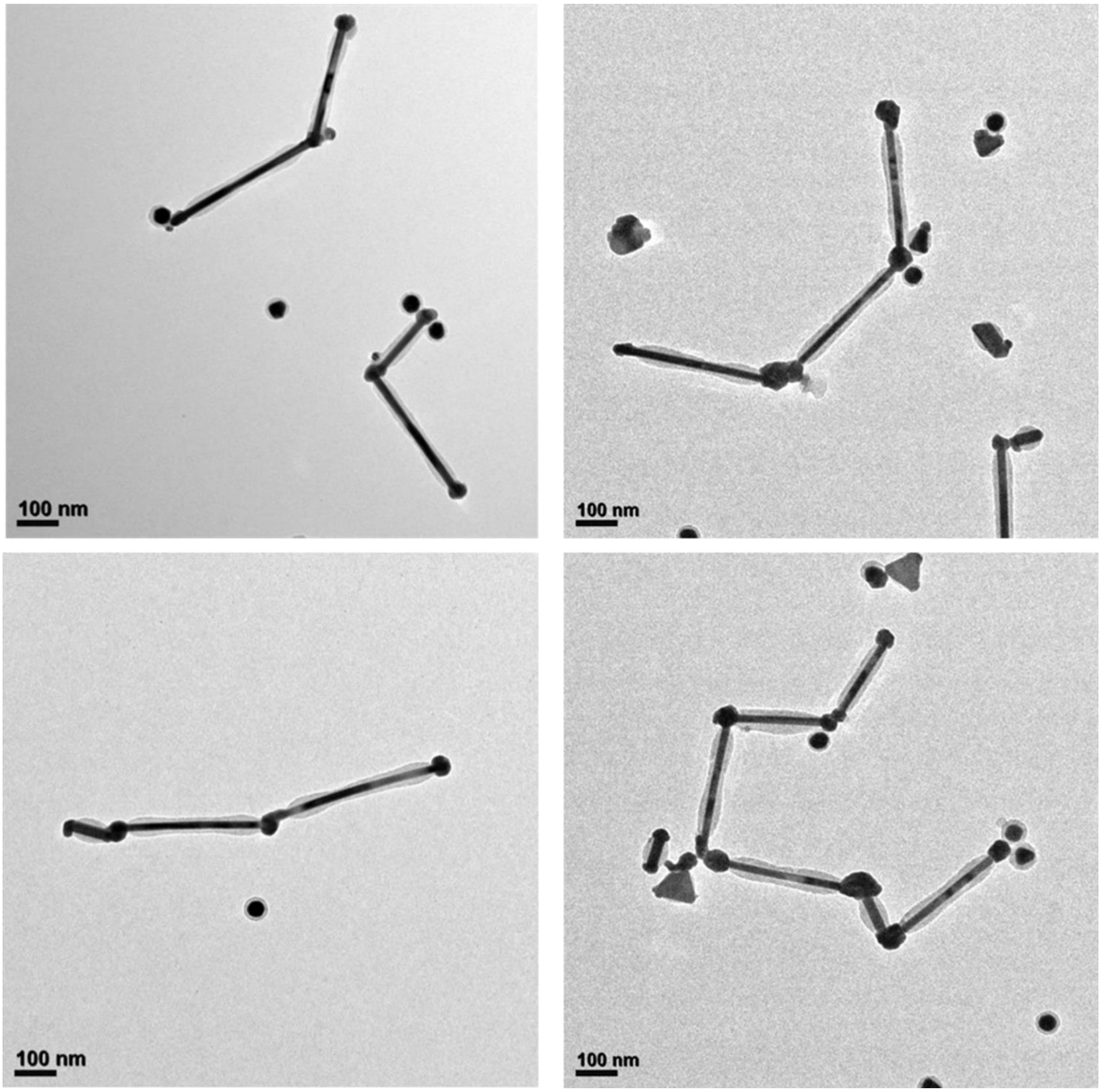

Supplementary Figure 33. Typical TEM images (high magnification) of the tip-to-tip coupling of AuNRs with Ag_2S as the junction.

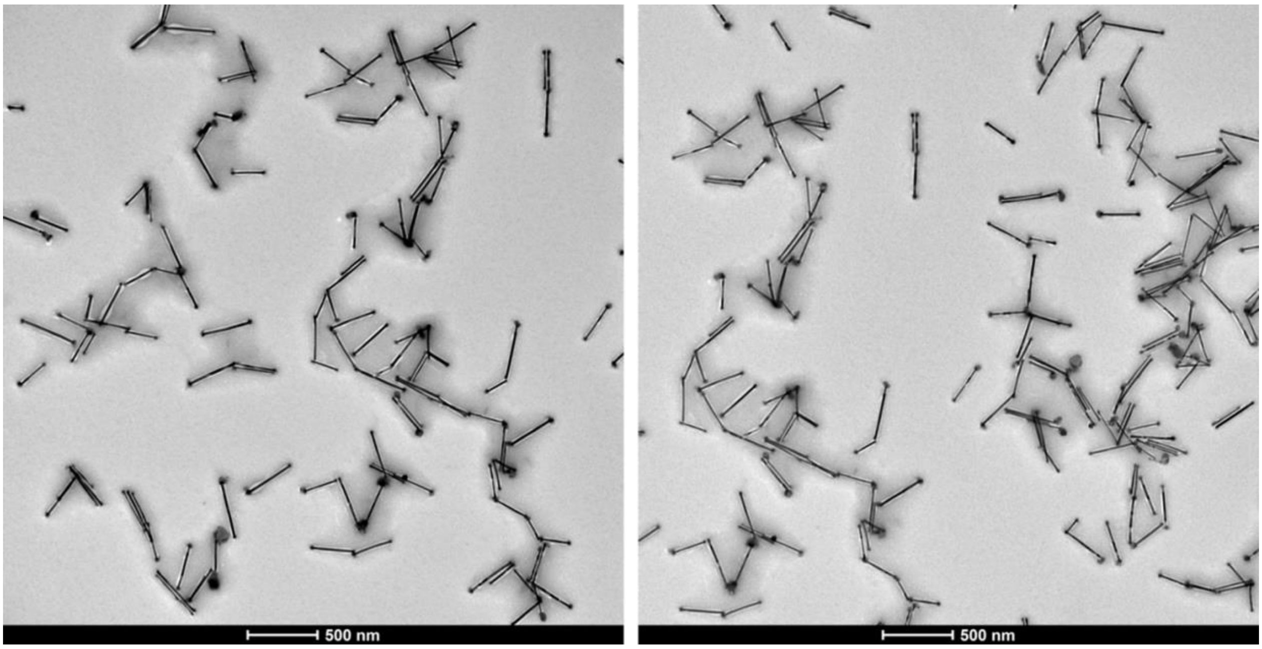

Supplementary Figure 34. Typical TEM images (low magnification) of the tip-to-tip coupling of AuNRs with Ag_2S as the junction. The tip-to-tip aggregation and the “welding” of the AuNRs can be observed by the TEM images (low magnification), which can be further confirmed by the following SAXS results.

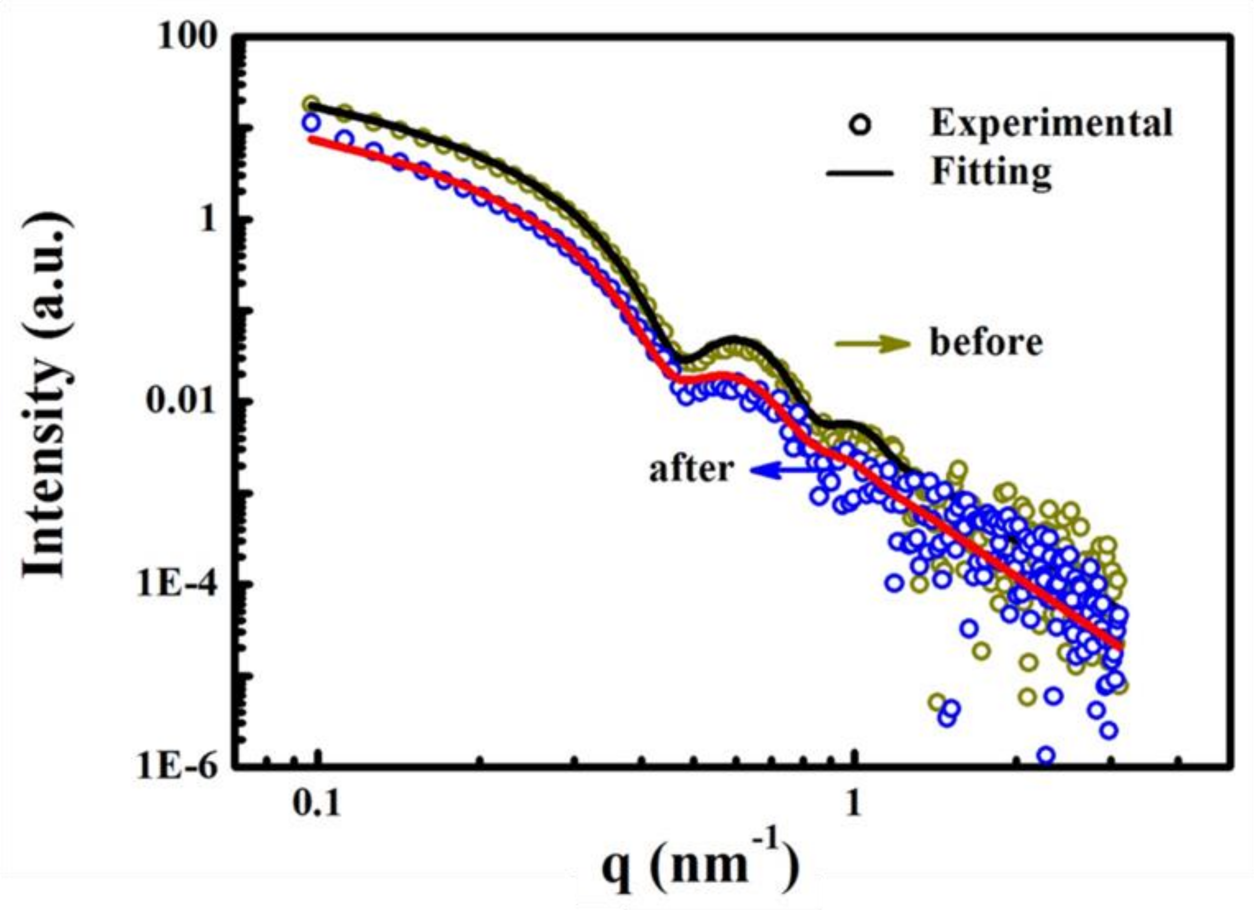

Supplementary Figure 35. Experimental (circles) and fitting (solid lines) SAXS curves before and after coupling.

Supplementary Discussion

Small angel X-ray scattering (SAXS) intensities were collected with an X-ray wavelength of 0.15412 nm in Xenoc-Nanoinxider as shown in **Supplementary Figure 35**. From the SAXS data, a SAXS-intensity vibration can be clearly observed and the vibration amplitude is damped with the increase of the q-vector. It appears that the scatterers in the samples are in a monodisperse system. Therefore, a cylinder model of the scatterers with narrow Gaussian distribution of the radius suggested by the TEM images was used to simulate the SAXS data. The simulated SAXS curves and the fitting parameters are shown in **Supplementary Figure 35** and **Supplementary Table 1**, respectively. For the as-synthesized AuNRs, the average diameter and length obtained by SAXS analysis (16 and 287 nm, respectively) match well with the size distribution obtained from TEM and SEM images (17.6 and 298 nm, respectively). Comparison of the SAXS results before and after sulfur-induced coupling showed that the average diameter of cylindrical particles remained

unchanged at 16 nm, whereas their average length increases from 297 to 488 nm after the coupling. The Gaussian distribution widths (σ) of the particles slightly increase from about 0.9 nm to 1.2 nm after coupling. Hence, the data agrees with the TEM observations in **Supplementary Figure 34**, where the coupling of the AuNRs leads to longer average length and wider length distribution.

Supplementary Table 1. SAXS fitting parameters with a cylinger model.

Samples	D (nm)	σ (nm)	L (nm)	B (nm^2)
Before	16.0	0.9	297.0	0.2
After	16.0	1.2	488.0	0.3

Note: D and L are the diameter and the length of the cylinder, respectively. σ is the Gaussian distribution widths of the cylinder-particle radii. B is the Porod correction parameter.

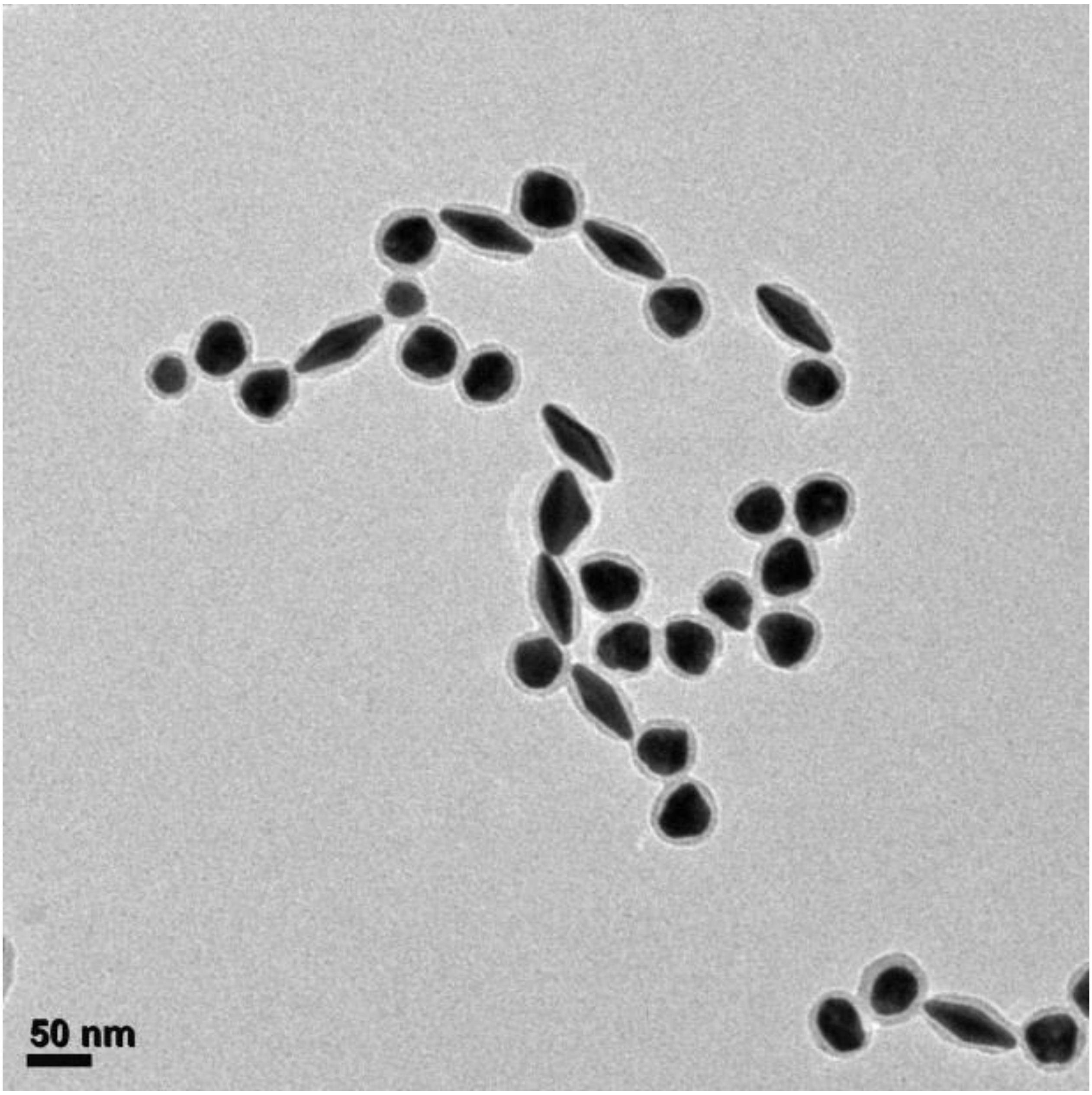

Supplementary Figure 36. TEM image of the pristine core-shell structure of (Au bipyramid-**1**)@PSPAA.

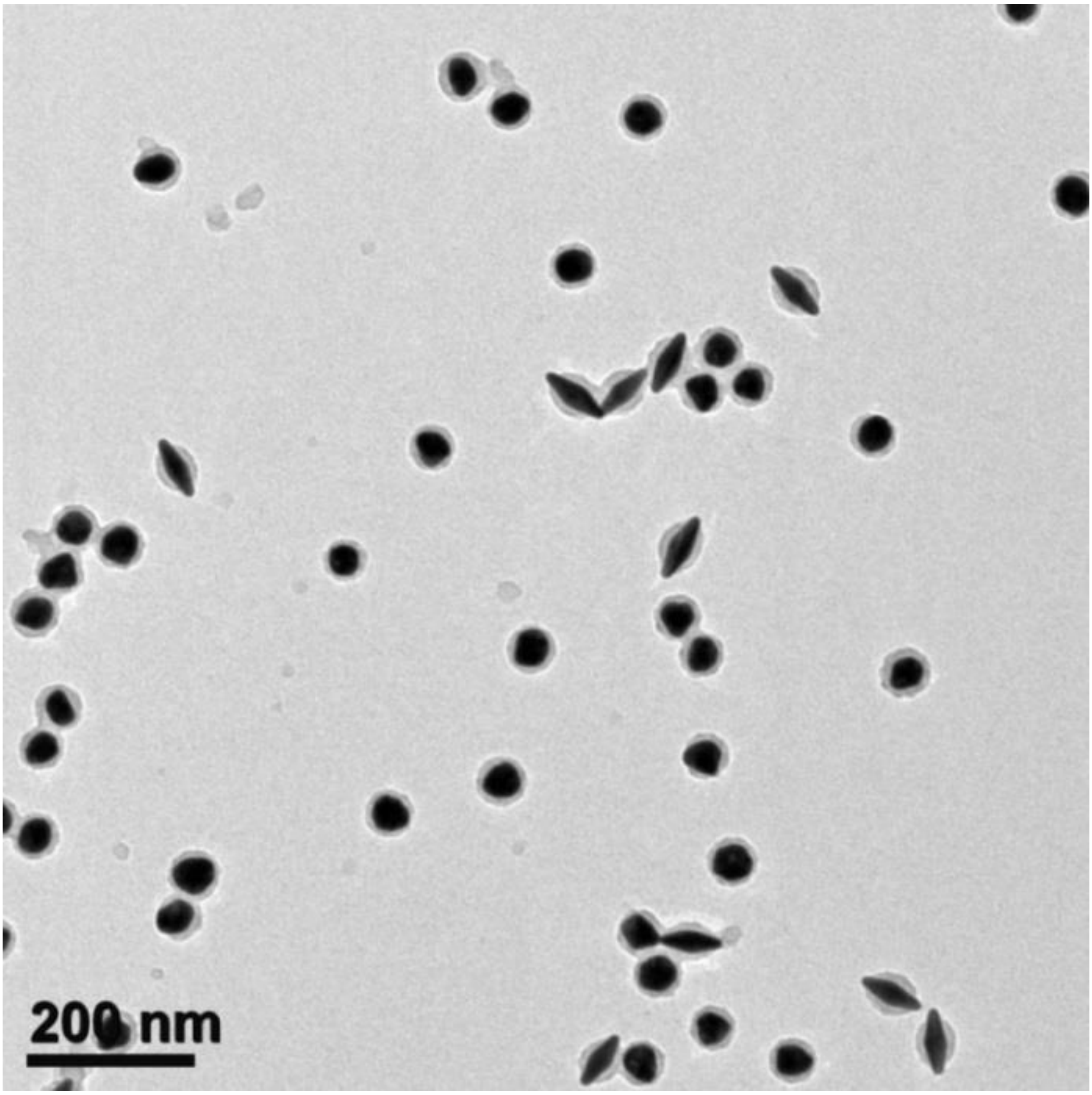

Supplementary Figure 37. TEM image of the (Au bipyramid-**1**)@PSPAA after being heated in water at 105 °C for 2 h.

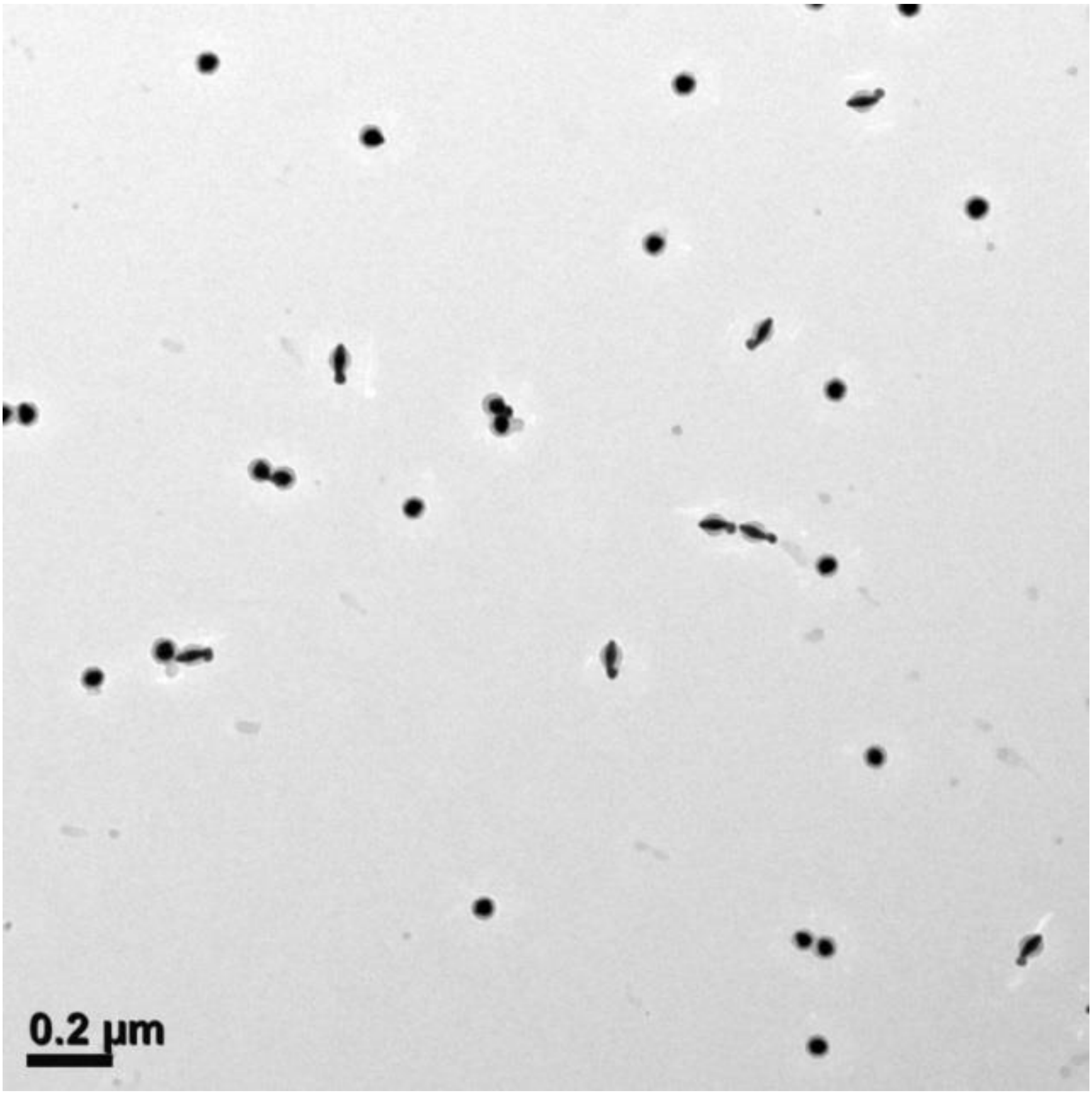

Supplementary Figure 38. TEM image of the transformed (Au bipyramid-**1**)@PSPAA with single Ag tip.

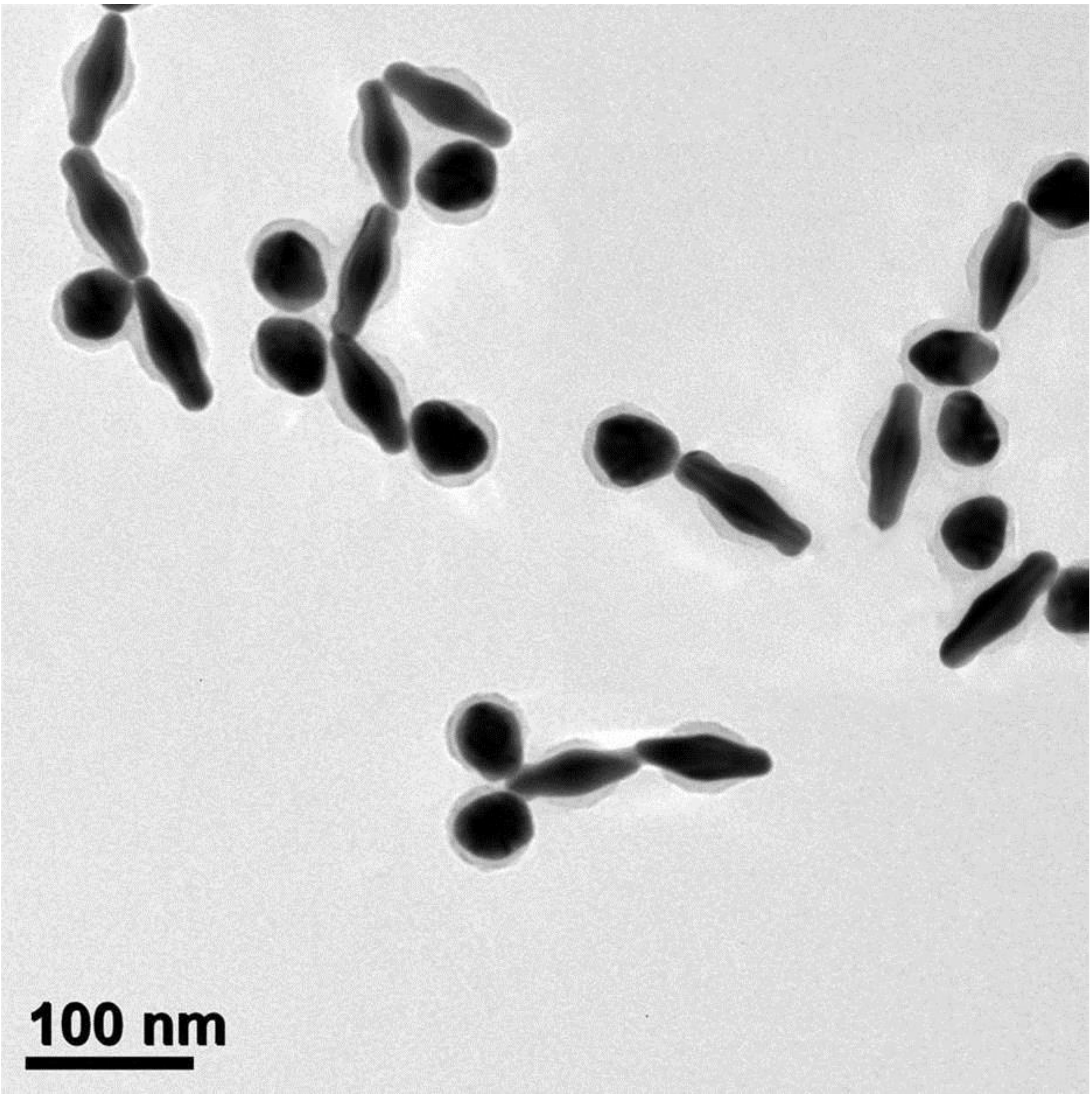

Supplementary Figure 39. TEM image of the transformed (Au bipyramid-**1**)@PSPAA with two Ag tips.

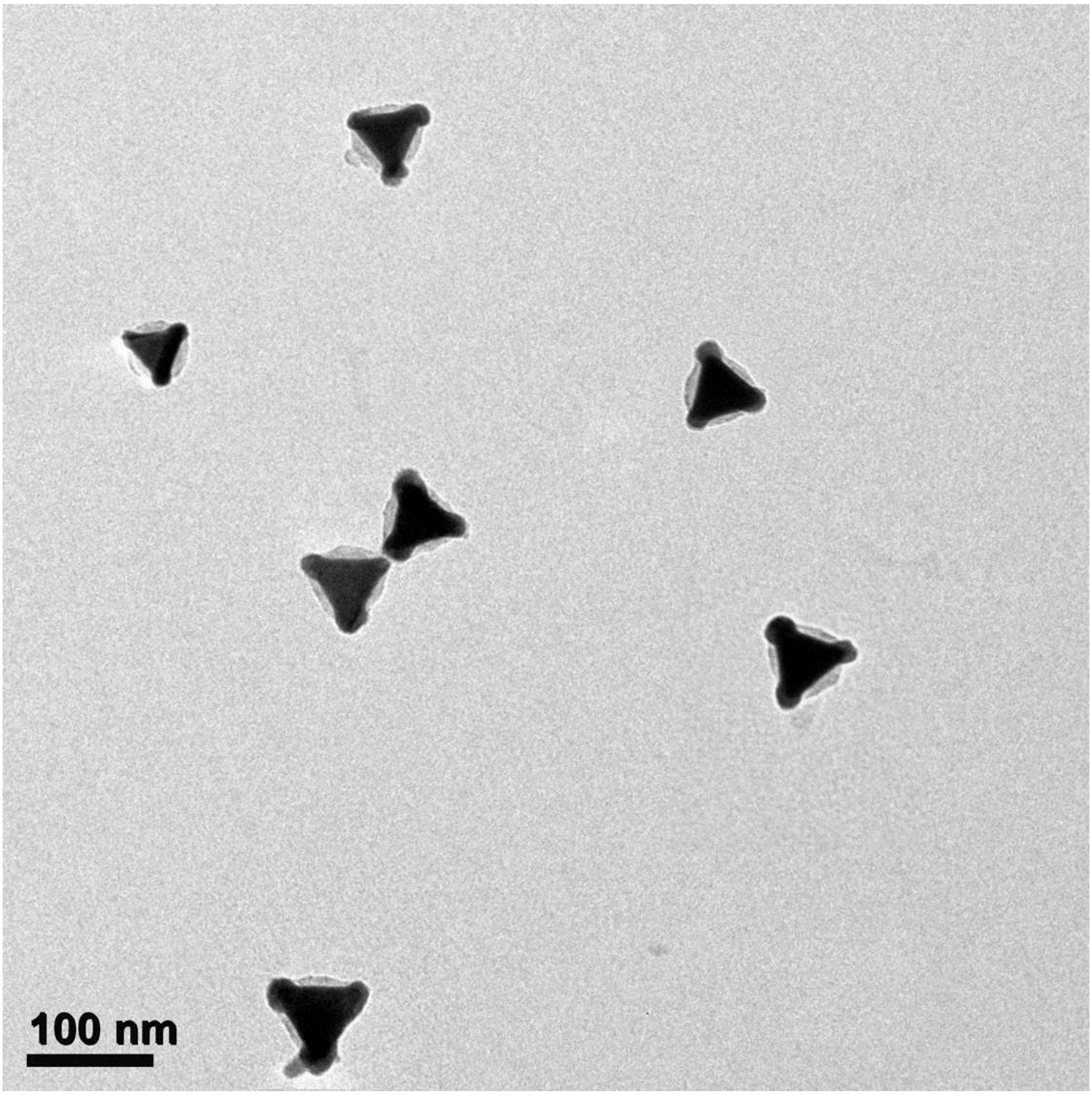

Supplementary Figure 40. TEM image of Ag deposition on transformed (gold triangular nanoprisms-**1**)@PSPAA.

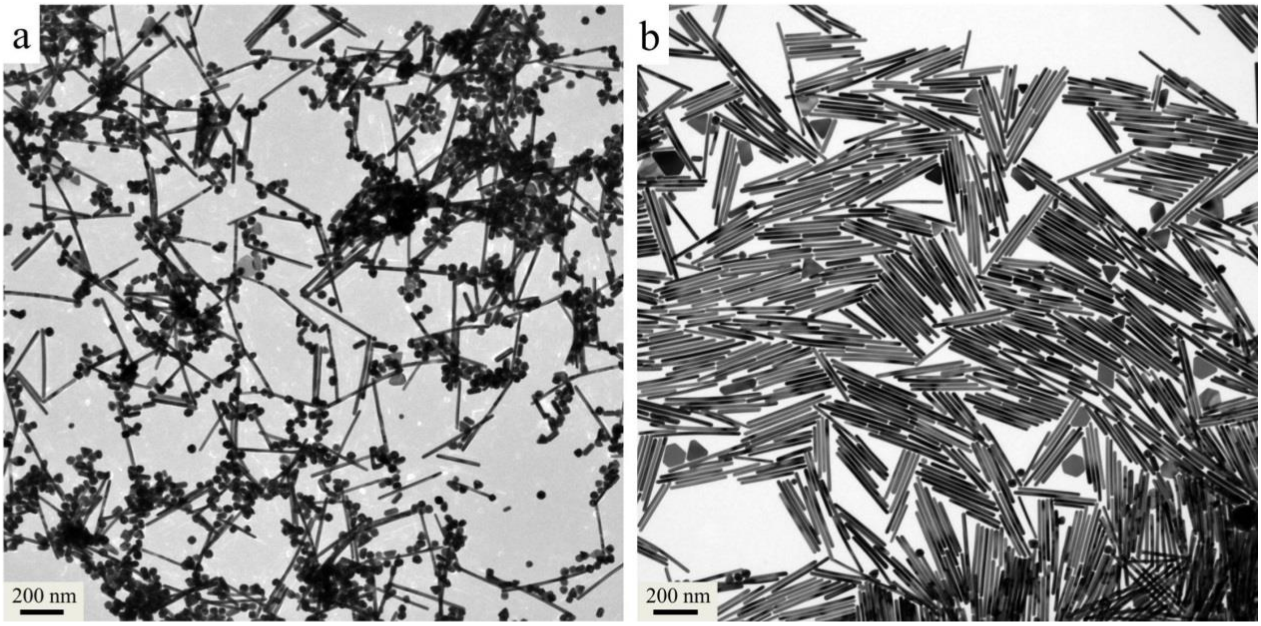

Supplementary Figure 41. TEM images of the AuNR before (a) and after (b) purification.

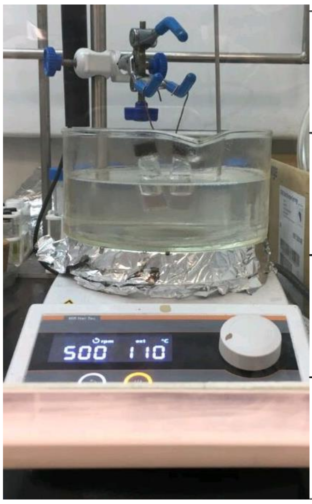

Temperature (°C)	105	110	115	120
Inner Pressure (kPa)	120.8	143.3	170.0	198.6
Atmospheric pressure (kPa)	101.3			
Net pressure (kPa)	19.5	42	68.7	97.3

Supplementary Figure 42. Experimental set-up in an airing chamber for the transformation of (AuNR-**ligand**)@PSPAA.
